# HaploSweep: Detecting and Distinguishing Recent Soft and Hard Selective Sweeps through Haplotype Structure

**DOI:** 10.1093/molbev/msae192

**Published:** 2024-09-17

**Authors:** Shilei Zhao, Lianjiang Chi, Mincong Fu, Hua Chen

**Affiliations:** Beijing Institute of Genomics, Chinese Academy of Sciences and China National Center for Bioinformation, Beijing 100101, China; School of Future Technology, University of Chinese Academy of Sciences, Beijing 100049, China; Beijing Institute of Genomics, Chinese Academy of Sciences and China National Center for Bioinformation, Beijing 100101, China; School of Future Technology, University of Chinese Academy of Sciences, Beijing 100049, China; Beijing Institute of Genomics, Chinese Academy of Sciences and China National Center for Bioinformation, Beijing 100101, China; School of Future Technology, University of Chinese Academy of Sciences, Beijing 100049, China; Beijing Institute of Genomics, Chinese Academy of Sciences and China National Center for Bioinformation, Beijing 100101, China; School of Future Technology, University of Chinese Academy of Sciences, Beijing 100049, China; CAS Center for Excellence in Animal Evolution and Genetics, Chinese Academy of Sciences, Kunming 650223, China

**Keywords:** selective sweep, soft sweep, adaptive evolution, haplotype structure

## Abstract

Identifying soft selective sweeps using genomic data is a challenging yet crucial task in population genetics. In this study, we present HaploSweep, a novel method for detecting and categorizing soft and hard selective sweeps based on haplotype structure. Through simulations spanning a broad range of selection intensities, softness levels, and demographic histories, we demonstrate that HaploSweep outperforms iHS, nSL, and H12 in detecting soft sweeps. HaploSweep achieves high classification accuracy—0.9247 for CHB, 0.9484 for CEU, and 0.9829 YRI—when applied to simulations in line with the human Out-of-Africa demographic model. We also observe that the classification accuracy remains consistently robust across different demographic models. Additionally, we introduce a refined method to accurately distinguish soft shoulders adjacent to hard sweeps from soft sweeps. Application of HaploSweep to genomic data of CHB, CEU, and YRI populations from the 1000 genomes project has led to the discovery of several new genes that bear strong evidence of population-specific soft sweeps (*HRNR*, *AMBRA1*, *CBFA2T2*, *DYNC2H1*, and *RANBP2* etc.), with prevalent associations to immune functions and metabolic processes. The validated performance of HaploSweep, demonstrated through both simulated and real data, underscores its potential as a valuable tool for detecting and comprehending the role of soft sweeps in adaptive evolution.

## Introduction

The elucidation of adaptive evolutionary mechanisms remains an essential question in the field of population genetics. Traditional methodologies have been established to identify selective sweeps by analyzing genomic polymorphisms, encompassing patterns such as allele frequency spectrum, haplotype structure, and cross-population allele frequency differentiation. These methods premised on the hard sweep paradigm, which posits that the beneficial mutations, newly arising or rare, are driven to predominance by natural selection, disrupting local polymorphism patterns in a distinctive manner that differs from neutral evolutionary processes due to the linkage disequilibrium among sites (Maynard Smith and Haigh 1974).

In the past decade, researchers in population genetics recognized that hard sweeps may not be the exclusive mode of adaptation in nature. This shift in perspective has emerged as an alternative, addressing the low probability of a beneficial allele arising and surviving random drift at low frequencies in early stages (Hermisson and Pennings 2005). Soft sweeps cover two primary scenarios, including selection acting on standing variation, which evolves under neutrality for some time; and recurrent beneficial mutations introduced multiple times during the sweep process. These mechanisms can result in multiple adaptive haplotypes at fixation.

The genetic polymorphism patterns surrounding sites undergoing soft sweeps, such as the allele frequency spectrum and genetic heterozygosity level, are more subtle than hard sweeps (Prezeworski et al. 2005; Barrett and Schluter 2008). Consequently, despite some methods like XP-CLR (Chen et al. 2010) retaining partial effectiveness in soft sweep detection, the majority of existing methods rooted in hard sweep models, have limited power to detect soft sweeps (Vatsiou et al. 2016).

The development of methodologies capable of effectively identifying soft sweeps and discerning them from hard sweeps presents a challenging yet crucial frontier in current research (Pritchard et al. 2010). Several methods have been proposed, falling into at least four categories: (i) Summary statistics, including H-statistics (Garud et al. 2015), G-statistics (Harris et al. 2018a), and nSL (Ferrer-Admetlla et al. 2014). The H-Statistics include multiple summary statistics measuring or combining frequencies of the first and second most abundant haplotypes, and have good power in detecting both soft and hard sweeps. The G-statistics are the genotype version of H-statistics. The nSL statistic, closely related to iHS, exhibits higher power in detecting soft sweep signals but lacks the ability to distinguish soft sweep from hard sweep; (ii) Probabilistic and maximum likelihood approaches, such as saltiLASSI and LASSI (Harris and DeGiorgio 2020; DeGiorgio and Szpiech 2022). LASSI uses a model of haplotype frequency spectrum distortion to detect sweeps and infer the number of actively sweeping haplotypes in a population. SaltiLASSI employs a composite likelihood approach to identify sweeps by searching for pronounced distortions in the spatial distribution of the haplotype frequency spectrum across the genome; (iii) Approximate Bayesian Computation (ABC) methods. Peter et al. (2012) adopted ABC framework to identify sweeps originating from either standing genetic variants or de novo mutations; (iv) Machine-learning (ML) methods, such as S/HIC, diploS/HIC, and evolBoosting, which aggregate summary statistics as informative sequence features for prediction (e.g. Tajima’s D, H12, etc.) and are trained with simulated data to discriminate hard sweeps, soft sweeps, and neutral regions (Pybus et al. 2015; Schrider and Kern 2016; Sheehan and Song 2016; Kern and Schrider 2018; Torada et al. 2019).

While these newly developed approaches offer valuable tools for exploring the impact of soft sweeps on phenotypic diversity and evolution in natural populations, some limitations remain. Harris et al. (2018b) noted that H-statistics may lose their ability to detect positive selection or differentiate hard and soft sweeps in complex nonequilibrium demographic histories. Additionally, Vy et al. (2017) found that calculating H-statistics with a fixed window size can be inefficient in distinguishing sweep types. Although ABC frame are flexible and extensively utilized in modeling, they become computationally expensive with the increased numbers of parameter and summary statistics. This is primarily due to the vast size of the parameter space and the inherent inefficiency of the rejection-sampling scheme. The identification of an appropriate subset of summary statistics for ABC poses a nontrivial task. Furthermore, ML methods require a substantial number of simulated data for classifier training, involving subjective decisions about data to simulate that may introduce bias towards preassumed scenarios. It is therefore crucial to test the robustness of the model on data simulated from alternative scenarios with different demographic models and other parameters. Further discussions on the limitations of these methods can be found in some recent reviews (Hejase et al. 2020; Rees et al. 2020).

## New Approaches

**Fig. 1.**
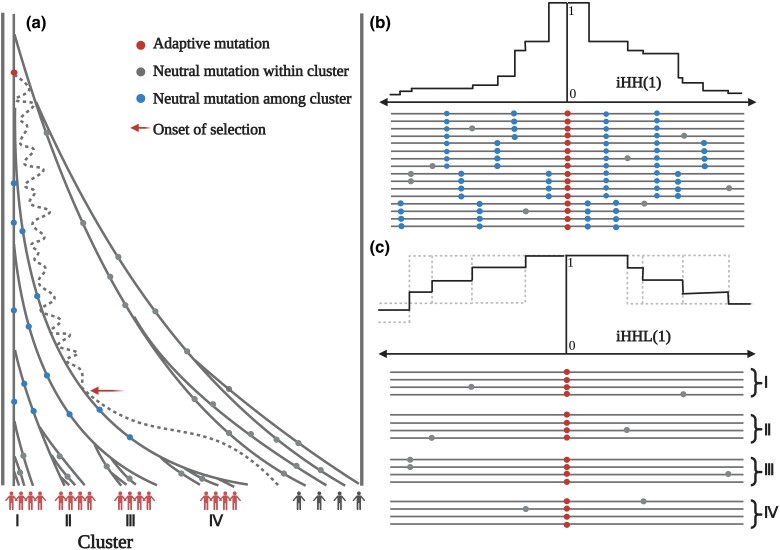
Schematic illustration of a soft sweep and the HaploSweep statistics. a) A genealogy of 20 haplotypes from a population undergone a soft sweep on a standing variation. The genealogy of 16 haplotypes carrying the beneficial allele traces back to a single mutational origin (denoted by the red dot) but multiple distinct founder haplotypes carrying this allele exist when positive selection sets in (denoted by the red arrow). b) iHH of haplotypes carrying the beneficial allele. Numerous mutations occurring before the onset of selection result in a rapid decrease in haplotype homozygosity. c) The average iHHL. The haplotypes are divided into different clusters, and iHH is calculated for each cluster separately. The dashed lines denote the EHH curve for each cluster, and the solid line denotes the average EHH curve. Haplotype homozygosity decreases slowly for local clusters, since only within-cluster homozygosity is included. The statistic iHSL is defined as the logarithmic ratio between iHHL(1) and iHHL(0). The statistic RiHS is defined as the logarithmic ratio between iHHL and iHH. Created in BioRender.

In this article, we present HaploSweep, a novel method designed to identify distinctive haplotype structure resulting from hitch-hiking effects during soft sweeps, distinguishing them from hard sweeps. HaploSweep builds on and advances the extended haplotype homozygosity (EHH) methods (Sabeti et al. 2002), adapting to the complexity of soft sweeps. As depicted in Fig. 1, during soft selective sweeps, the haplotypes carrying the beneficial alleles can be traced back to multiple ancestral founding haplotypes. The diversity among these ancestral founding haplotypes leads to a rapid decline in EHH statistic for the haplotypes carrying the beneficial allele and are not significantly different from haplotypes under neutrality. Consequently, traditional EHH-based methods, like iHS, have limited efficacy in detecting soft sweeps.

Taking into account the multifounder clustering characteristic of haplotypes carrying the beneficial allele during soft selective sweeps, we propose novel statistics, integrated haplotype homozygosity for local clusters (iHHL) and a logarithmic ratio variant (iHSL). Haplotypes are grouped into distinct clusters, and iHH (integrated haplotype homozygosity) is calculated separately for each cluster (Voight et al. 2006). Within each cluster, the decline of haplotype homozygosity occurs at a slower rate. Consequently, we anticipate observing higher values of iHHL under soft sweeps compared to neutral evolution. iHSL is defined as the logarithmic ratio between iHHL(1) and iHHL(0). Here, “1” in parentheses represents the derived allele and “0” denotes the ancestral allele (refer to the HaploSweep statistics iHSL and RiHS in Materials and Methods section for further details). This statistic enables the identification of both hard and soft sweeps. In addition, we introduce RiHS, which represents the logarithmic ratio between iHHL and iHH, to facilitate the classification of sweep types.

Through extensive simulations, we demonstrate HaploSweep’s adeptness across a spectrum of demographic histories, selection intensities, and softness levels. Moreover, simulations show that HaploSweep achieves remarkable accuracy in sweep classification. Utilizing HaploSweep on data from the 1000 Genomes Project uncovers novel candidate genes potentially vital to human adaptation. Our methods’ performance in both simulation and empirical data highlights its significant role in detecting and understanding the role of soft sweeps in adaptive evolution.

## Results

### Performance on Simulated Data

We first evaluate the performance of HaploSweep using simulation data under various demographic models (supplementary fig. S1, Supplementary Material online).

In the first scenario, we simulate a stable diploid population with a constant size of 10,000 individuals. Two types of soft sweeps arising from standing genetic variation (SGV) and recurrent mutation (SRM) are simulated. For SGV, we investigate different initial allele frequencies of 0.05, 0.1, and 0.2, while for SRM, we vary mutation rates using *θ* values of 5, 10, and 50.

**Fig. 2.**
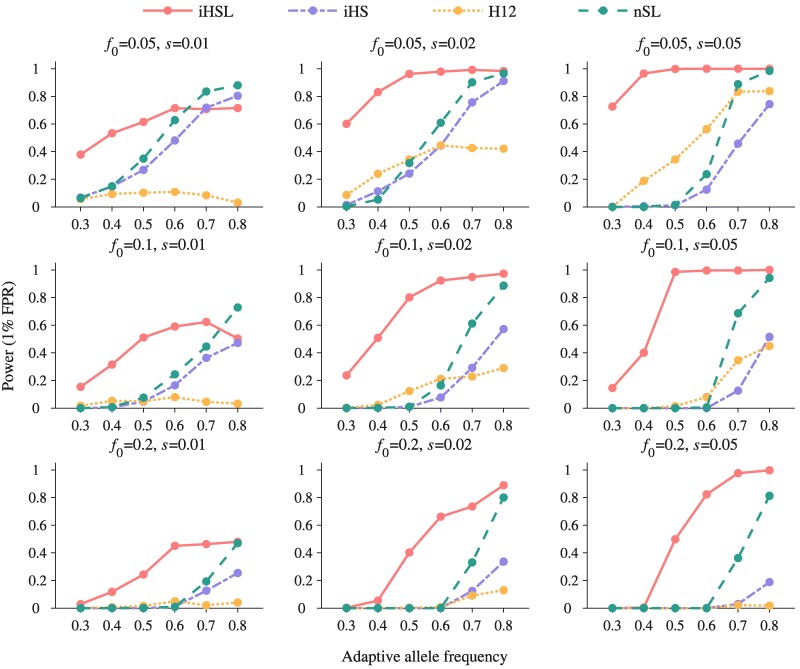
Comparison of the detection power of four methods for detecting soft selective sweeps from standing variations across a range of initial allele frequencies $f_{0}$ and selection intensities *s*. Power, defined as the proportion of simulations rejecting the neutral null hypothesis at a 1% FPR, is plotted. The *x*-axis reflects the adaptive allele frequencies observed in the contemporary population samples. Different methods are distinguished by unique lines colors and styles. Here, H12 is calculated with a window size of 1,001 SNPs, the optimal window size among seven candidate window sizes, as shown in supplementary fig. S3, Supplementary Material online.

**Fig. 3.**
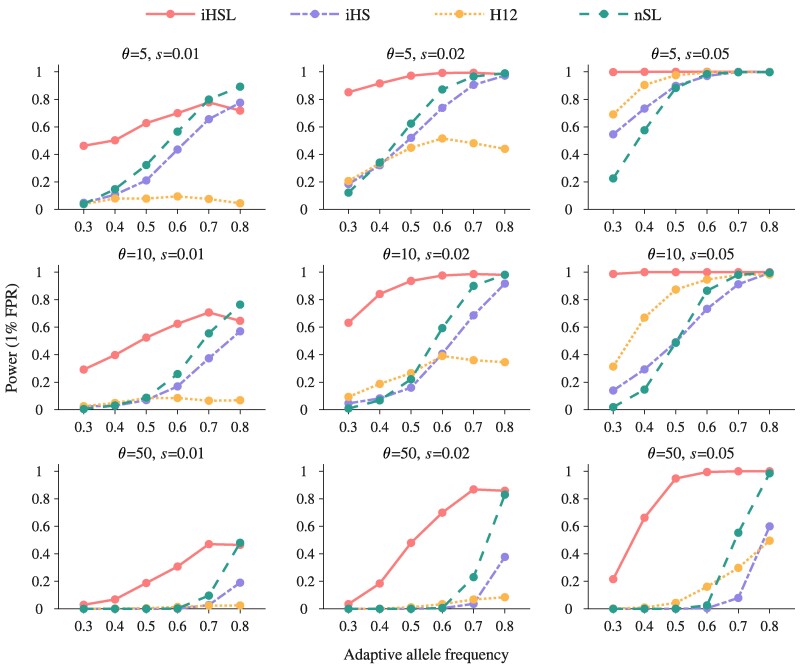
Comparison of the detection power of four methods in detecting soft selective sweeps from recurrent mutation across a range of scaled mutation rate $\theta =4N_{e}\mu$ and selection intensities *s*. Power, defined as the proportion of simulations rejecting the neutral null hypothesis at a 1% FPR, is plotted. The *x*-axis reflects the adaptive allele frequencies observed in the contemporary population samples. Different methods are distinguished by unique lines colors and styles. Here, H12 is calculated with a window size of 1,001 SNPs, the optimal window size among seven candidate window sizes, as shown in supplementary fig. S4, Supplementary Material online.

To assess the ability of HaploSweep to detect sweep signals, we compare its performance to three commonly used methods: iHS, H12, and nSL. We analyze their effectiveness across varying levels of softness ($f_{0}=0.05$, 0.1, 0.2 or *θ* values of 5, 10, 50), selection intensities (s=0.01, 0.02, 0.05), and adaptive allele frequencies (ranging from 0.3 to 0.8) while maintaining a 1% false-positive rate (FPR). Remarkably, HaploSweep excels in identifying soft sweeps in both SGV and SRM scenarios, particularly when the soft sweeps are with a high degree of softness (Figs. 2 and 3). For example, with an $f_{0}$ of 0.2, HaploSweep achieves a mean power of 0.4349 at a 1% FPR, outperforming iHS (0.0594), H12 (0.0224), and nSL (0.1655). Similarly, for SRM with an *θ* of 50, HaploSweep demonstrates a mean power of 0.5261, surpassing iHS (0.0733), H12 (0.0709), and nSL (0.1783). Notably, nSL performs best in the case of hard sweeps, with a mean power of 0.9929 (supplementary fig. S2, Supplementary Material online and supplementary table S1, Supplementary Material online).

While originally designed to detect hard sweeps, iHS also demonstrates some capability in detecting soft sweeps with mild to moderate softness. To elaborate further, we generate EHH curves for both iHS and HaploSweep, illustrated in supplementary figs. S5–S7, Supplementary Material online, displaying the EHH curve for ten repeated simulations with selection intensities set at 0.01, 0.02, and 0.05, and mutation rates at 5, 10, and 50. In cases of soft sweeps with mild to moderate softness, the EHH curve of haplotypes containing the adaptive allele exhibits a rapid decline followed by an extended tail. The rapid decline is attributed to the initial heterogeneity among different founder haplotype groups, and the prolonged tail is a result of the homozygosity of haplotype within the same group. However, as the number of founder haplotype increases, the tail diminishes until it becomes indistinguishable from neutrality. HaploSweep overcomes this issue by separating haplotype groups when calculating EHH.

To further explore the performance of HaploSweep under weaker selection pressures, we perform simulations with selection intensities of s=0.001, 0.002, and 0.005. These simulations are implemented in the same manner as in SRM. All four methods exhibit limited power in detecting soft sweep signals with such weak selection intensities (supplementary fig. S8, Supplementary Material online), with HaploSweep generally demonstrating the highest efficacy. The mean power across the weak selection intensities range is 0.0936 for HaploSweep, 0.0419 for iHS, 0.0124 for H12, and 0.0547 for nSL.

To assess the effect of sample size on the performance of HaploSweep, we perform two sets of simulations with sample sizes of 100 and 50 haplotypes, respectively. The simulation parameters are the same as those in SRM, except for the reduced sample size. For simulations with 100 haplotypes, the mean powers are as follows: HaploSweep iHSL 0.3539, iHS 0.2753, H12 [using an optimal window size of 401 single nucleotide polymorphisms (SNPs)] 0.1714, and nSL 0.3424 (supplementary fig. S9, Supplementary Material online). For simulations with 50 haplotypes, the mean powers are: HaploSweep iHSL 0.2359, iHS 0.1913, H12 (using a window size of 201 SNPs) 0.1036, and nSL 0.2597 (supplementary fig. S10, Supplementary Material online). All four methods tested demonstrate decreased power as the sample size decreased.

**Fig. 4.**
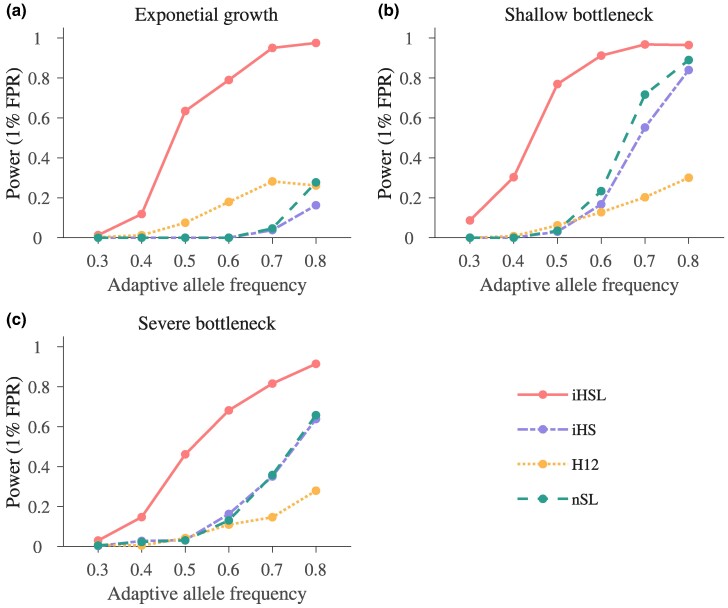
Comparison of the detection power of four methods for detecting soft sweep signals from standing variations under different demographic models. Selection intensity is set to s=0.02, and the initial allele frequency is set to $f_{0}=0.1$. a) Exponential growth model. The effective population grows from 1,000 to 20,000 starting 1,000 generations ago. b) Mild bottleneck model. The effective population size is reduced to half of the normal value, and the bottleneck lasts for 400 generations. c) Severe bottleneck model. The effective population size is reduced to 0.1 of the normal value, and the bottleneck lasts for 400 generations. Details of the demographic models are shown in supplementary fig. S1, Supplementary Material online. The optimal window size of H12 for all three simulations is set to be 1,001 SNPs, chosen among seven candidate window sizes: 21, 51, 101, 201, 401, 1,001, and 2,001 SNPs.

We also conduct simulations under three nonequilibrium demographic scenarios: exponential growth, moderate bottleneck, and severe bottleneck (refer to the Coalescent simulations section and supplementary fig. S1, Supplementary Material online for detailed information). In all of these scenarios, HaploSweep consistently outperforms iHS, H12, and nSL (refer to Fig. 4). The mean power values clearly illustrate the superiority, with HaploSweep achieving a mean power of 0.5854, compared to 0.1669 for iHS, 0.1168 for H12, and 0.1891 for nSL (see supplementary table S2, Supplementary Material online).

### Performance of HaploSweep in Pinpointing the Adaptive Locus

To evaluate how effectively HaploSweep pinpoints the adaptive locus, we calculate the peak distance and rank distributions of the adaptive locus. The peak distance measures the distance between the peak signal and the actual adaptive locus, while rank indicates the position of the adaptive locus in a sorted list based on the significance of sites. The peak distance and rank distributions are generated separately for hard and soft sweeps (recurrent mutation, θ=5,10). We employ a constant demographic model as in Fig. 3, with selection intensity s=0.02. The iHSL values spanning 500 kb upstream to 500 kb downstream of the adaptive locus are calculated for 1,000 simulation replicates to obtain these metrics.

As shown in supplementary fig. S11, Supplementary Material online, the peak distance and rank distributions exhibit distinct shapes between hard and soft sweeps. The distributions for hard sweeps are J-shaped, whereas those for soft sweeps are L-shaped with a long tail. This disparity likely stems from the differing levels of linkage disequilibrium induced by the two types of selective sweeps. Furthermore, the shapes of these distributions can be influenced by several factors, such as selection intensities, selection onset times, softness, and sample sizes.

### Comparison of the Power of *iHSL* and $iHSL^{2}+RiHS^{2}$ in Detecting Soft Sweeps

To explore the relationship between iHSL and RiHS, we compute the Pearson correlation coefficient (*ρ*) and *P*-values in six datasets: three from the 1000 Genomes Project (1KGP): CHB (Han Chinese from Beijing), CEU (Utah residents with Northern and Western European ancestry), and YRI (Yoruba from Nigeria) and their respective neutral simulated counterparts (details in Human out of African demographic model section). The results are presented in supplementary fig. S12, Supplementary Material online. Pearson’s correlation coefficients are less than 0.004 for the three simulated data sets with *P*-values >0.1 (supplementary fig. S12A–C, Supplementary Material online). For all three real datasets, the Pearson’s correlation coefficients are smaller than 0.02 (CHB ρ=0.0022, CEU ρ=0.0029, YRI ρ=0.0195), with *P*-values below $10^{-6}$ (supplementary fig. S12D–F, Supplementary Material online). We introduce the composite statistic $\mathrm{RiHSL}={\mathrm{iHSL}}^{2}+{\mathrm{RiHS}}^{2}$. In simulated neutral data, the distribution of RiHSL fits well with a Chi-Square distribution with two degrees of freedom, whereas this fit is less precise for real data. We hypothesize that the significant correlation between iHSL and RiHS, along with the small values of correlation coefficients observed in the real data, may be influenced by the relatively small proportion of sites under selective sweeps.

We compare the power of iHSL and RiHSL in detecting soft selective sweeps across different demographic models with an initial allele frequency of $f_{0}=0.1$ and selection intensity of s=0.02. In the analysis depicted in supplementary fig. S13, Supplementary Material online, RiHSL exhibits higher power than iHSL in simulations with a low beneficial allele frequency, but shows lower power in simulations with a high beneficial allele frequency. Users of HaploSweep have the option to access both iHSL and RiHSL values for their analysis.

### Robustness Against Variation of Recombination Rate and Mutation Rate

We evaluate the robustness of HaploSweep, iHS, nSL, and H12 against fluctuations in recombination and mutation rates by examining their FPRs under varying rates. The results illustrated in supplementary fig. S14, Supplementary Material online indicate that H12 shows an increased FPR with lower recombination rates and higher mutation rates, aligning with the observations of Vy et al. (2017). Across all recombination rates, the mean FPR values are 0.1030 for H12, 0.0063 for HaploSweep, 0.0094 for iHS, and 0.0079 for nSL.

HaploSweep, iHS, and nSL employ haplotypes carrying two alleles as internal controls for local fluctuations in mutation and recombination rates (Voight et al. 2006; Field et al. 2016). This strategy contributes to the high robustness of HaploSweep, iHS, and nSL against variations in recombination and mutation rates.

### The Effect of the Density of Background Neutral Variation on the Performance of HaploSweep

To investigate the impact of background neutral variation density on the performance of HaploSweep, we conduct simulations with mutation rates ranging from $0.5\times 10^{-8}$ to $2.5\times 10^{-8}$ per bp per generation, using the equilibrium demographic model described in the Coalescent simulation section. We set the intensity of selection at s = 0.01, the frequency of the beneficial allele in the contemporary population sample at f=0.5, and the scaled mutation rate of the beneficial allele to θ=10. Simulations of neutral regions are also conducted with the same background mutation rates. The distribution of iHSL values for soft selective sweeps under different mutation rates is shown in supplementary fig. S15, Supplementary Material online. The mean values of normalized iHSL are 2.3138, 2.4470, 2.3698, 2.4497, and 2.4091 for mutation rates *μ* of $0.5\times 10^{-8}$, $1\times 10^{-8}$, $1.5\times 10^{-8}$, $2\times 10^{-8}$, and $2.5\times 10^{-8}$, respectively. The power at a 1% FPR is 0.5150, 0.5436, 0.5401, 0.5360, and 0.5245, respectively.

A two-sample t-test reveals that the means of the iHSL values for simulations with *μ* of $0.5\times 10^{-8}$ and $2.5\times 10^{-8}$ are significantly different (P-value=0.0367), while no significant differences are found for $1\times 10^{-8}$, $1.5\times 10^{-8}$, and $2\times 10^{-8}$ compared to $2.5\times 10^{-8}$ (P-values=0.4176, 0.3762, and 0.3869, respectively). In summary, the performance of HaploSweep shows a slight reduction when the density of background neutral variation is considerably low.

### Distinguishing Soft and Hard Sweeps

**Fig. 5.**
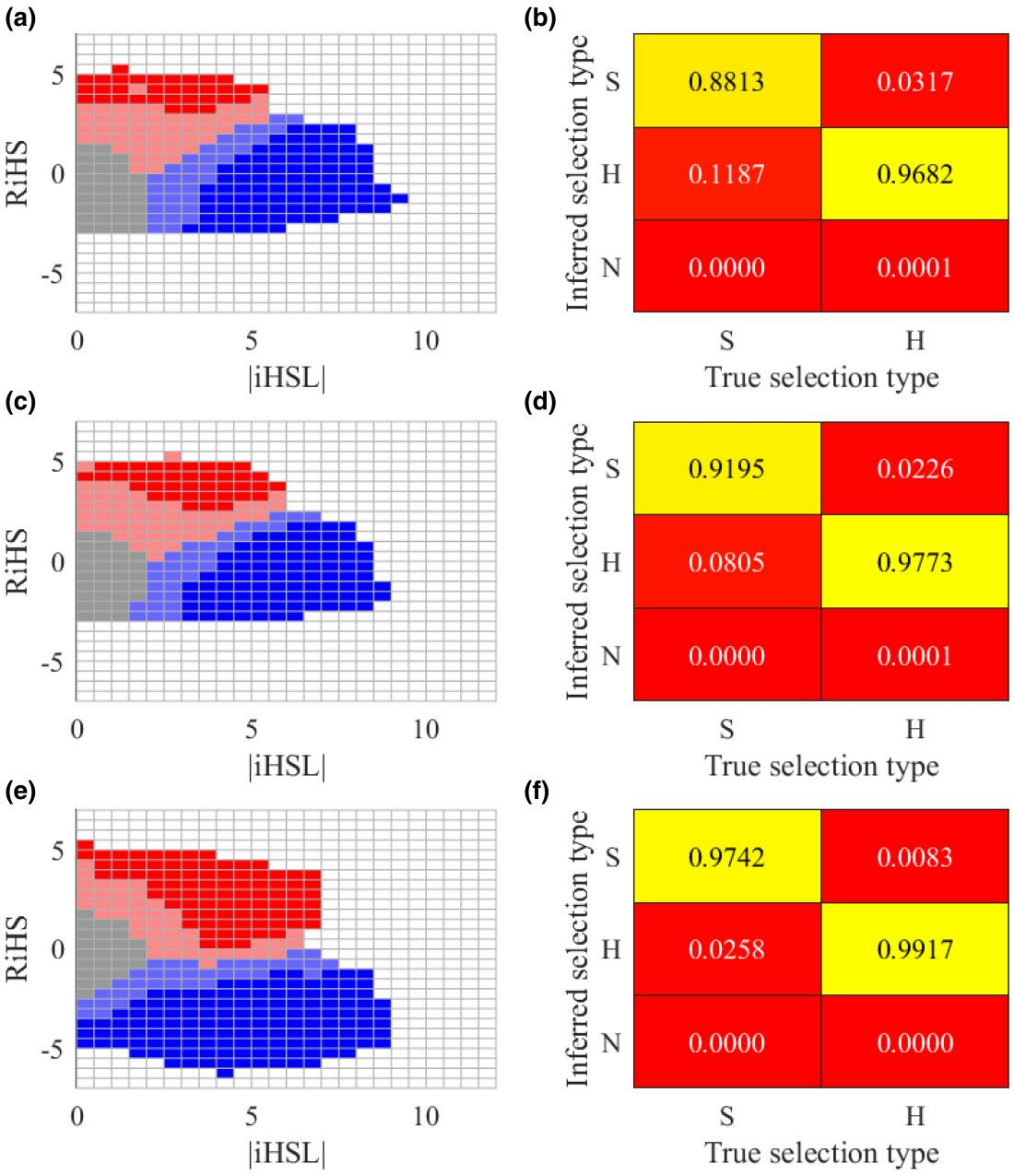
The distribution of RiHS and iHSL values for different sweep types and the ability to distinguish between soft sweeps and hard sweeps. In this context, “N” represents neutral, “H” represents hard sweep, and “S” represents soft sweep. a, c, e) The distribution of RiHS and iHSL values for simulation data of CHB, CEU, and YRI, respectively is shown. The grid in dark red denote region with > 95% simulation data points under soft sweep. The grids in light red denote the region with the majority of the simulation data type as soft sweep. Dark and light blue grids for hard sweeps are defined similarly. The gray grids denote the region with the mode of the simulation data type as neutral. b–d) The ability to distinguish soft and hard sweeps for simulation data of CHB, CEU, and YRI, respectively are shown.

We investigate the ability of HaploSweep to distinguish soft and hard sweeps by analyzing the simulated distribution of HaploSweep values for both soft and hard sweeps across varying selection intensities, selection onset times, and initial adaptive allele frequencies (details can be found in Human out-of-African demographic model section). The simulated distributions of HaploSweep values for the CHB, CEU, and YRI populations are depicted in Fig. 5a, c, and e. In practice, we are more concerned with the sweep type of significant signals. Therefore, we calculate the confusion matrices for soft and hard sweeps with a P-value<0.01 (Figs. 5b, d, and f). The complete confusion matrices can be found in supplementary fig. S16, Supplementary Material online. Notably, HaploSweep achieves high classification accuracies for 0.9247, 0.9484 for CEU, and 0.9829 for the YRI populations. Particularly noteworthy is that HaploSweep performs best in distinguishing sweep types within the YRI population. This observation is expected given the recent bottleneck experienced by the CHB and CEU populations during their migration out of Africa, which reduced the number of founder haplotypes in contemporary genome samples and made discerning soft sweeps from hard sweeps more challenging for these populations.

We compare the performance of HaploSweep, H-statistics, and diploS/HIC in classifying sweep types. We calculate H-statistics on simulation datasets of CHB, CEU, and YRI using the out-of-Africa demographic model identical to that used for HaploSweep. H-statistics are still calculated using the optimal window size chosen from a range of sizes (21, 41, 101, 201, 401, 1,001, and 2,001 SNPs). We also train and test diploS/HIC using the same out-of-Africa demographic model. Furthermore, we compared the performance of HaploSweep, H-statistic, and diploS/HIC in classifying sweep types using simulated data with smaller sample sizes of 100 and 50 haplotypes. The selection parameters are identical for the three parameters, and only partial sweeps are used to generate the confusion matrices for consistency. As shown in supplementary fig. S16, Supplementary Material online, HaploSweep outperforms H-statistics and diploS/HIC in classifying sweep types with higher accuracy (mean values of the diagonal of the confusion matrix) across all simulation scenarios.

Additionally, we explore the impact of inaccurate demographic information on identifying sweep types. By employing the simulated distribution from the CHB population to classify sweep types in the CEU population, we achieve an accuracy of 0.9367 for significant sweep signals with a P-value<0.01. Similarly, using the simulated distribution obtained from the CEU population for classifying sweep types in the CHB population results in an accuracy of 0.9323 (supplementary fig. S17, Supplementary Material online). These results demonstrate the robust classification ability of HaploSweep even in the presence of uncertainties in population history.

### The Identification of Soft Shoulder

**Fig. 6.**
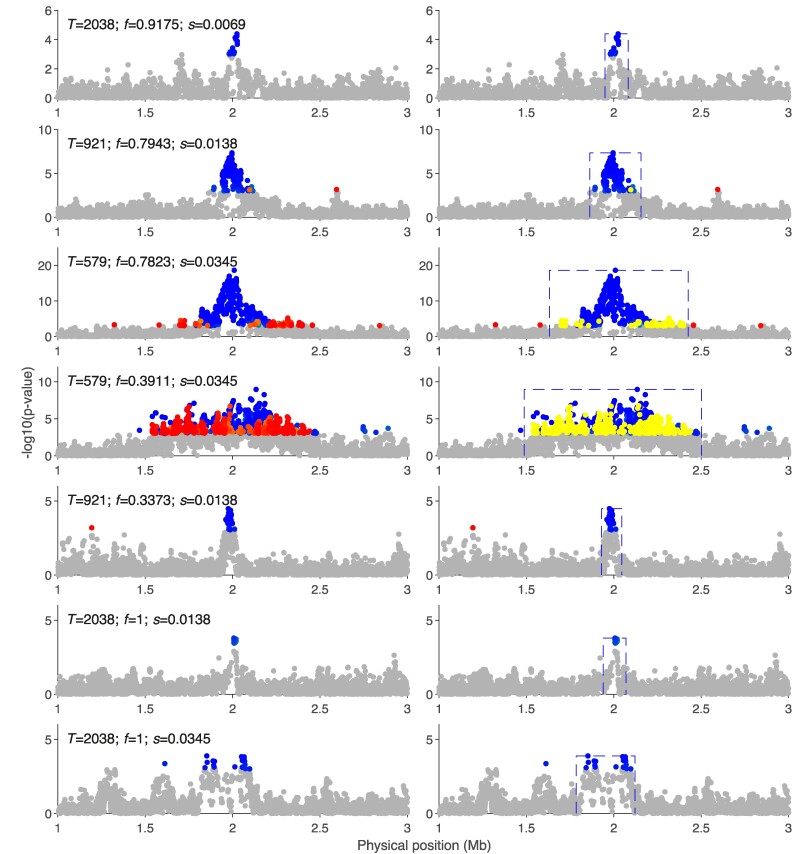
A strategy to identify soft shoulder under a hard sweep with a range of parameters. *T* denotes the time of selection onset. Panels on the left: gray dots indicate SNPs with HaploSweep *P*-values greater than 0.001, while blue and red dots denote SNPs identified as undergoing hard sweeps and soft sweeps, respectively. Panels on the right: Blue boxes indicate a single region of soft shoulder affected by a selective sweep. Yellow dots depict the SNPs affected by soft shoulder and sweep types are revised by the correction strategy.

The concept of soft shoulder, as proposed by Schrider et al. (2015), suggests that hard sweeps may exhibit patterns akin to soft sweeps and partial sweeps at nearby genetic loci. To investigate the impact of the soft shoulder effect on HaploSweep, we conduct simulations using extended 4Mb segments with the beneficial allele positioned at the center under a hard sweep. Simulations are carried out with various selection intensities and onset times under the demographic history of CHB (see the Coalescent simulation section). The results, presented in Fig. 6, illustrate the sweep signals and the sweep types identified by HaploSweep. In the figure, gray dots indicate SNPs with HaploSweep *P*-values exceeding 0.001, while blue and red dots denote SNPs identified as undergoing hard sweeps and soft sweeps, respectively.

In the left panels of Fig. 6, it is observed that the flank region of a recent strong hard sweep tends to be classified as a soft sweep by HaploSweep. Nonetheless, the leading significant SNPs in the same region are correctly identified as part of a hard sweep. To address the misclassification of soft shoulders, we devise a strategy. For each target SNP, we calculate the proportion of significant SNPs surrounding it. If the proportion exceeds a predefined threshold, the target SNP is designated as a significant core SNP. Subsequently, a single sweep region is defined as the contiguous stretch covered by consecutive significant core SNPs. The type for a single sweep is determined by the leading SNP with the most significant *P*-value. The identified sweep regions are highlighted in the right panels of Fig. 6, and SNPs with revised sweep types are depicted as yellow dots. This strategy effectively identifies sweep regions and rectifies the sweep type influenced by the soft shoulder effect, as demonstrated in Fig. 6.

### Soft Selective Sweeps Identified in Three Populations of the 1 KG Project

We perform a genome-wide analysis to detect recent selective sweeps in three populations from the 1000 Genomes Project (1KGP): CHB (Han Chinese from Beijing), CEU (Utah residents with Northern and Western European ancestry) and YRI (Yoruba from Nigeria), consisting of 103, 99, and 108 unrelated individuals, respectively. The genome-wide *P*-values obtained from HaploSweep, iHS, and nSL are depicted in Manhattan plots (refer to supplementary figs. S18–S20, Supplementary Material online). Our analysis identifies 205, 253, and 307 genomic regions experiencing soft selective sweeps in CHB, CEU, and YRI, with 299, 344, and 629 genes corresponding to population-specific soft sweeps for each group. In contrast, regions with signals of hard selective sweeps numbered 102, 81, and 153 in CHB, CEU, and YRI, with genes under population-specific hard selective sweeps totaling 281, 199, and 152 for CHB, CEU, and YRI.

To further understand the biological implications of these genes, we perform a gene set enrichment analysis focused on population-specific soft sweeps using KOBAS-i (Bu et al. 2021). Detailed results are presented in Table 1, with complete lists of enrichment terms provided in supplementary tables S3–S11, Supplementary Material online. Notably, terms related to immune response and metabolism are prominently enriched across populations. The lipid metabolism pathway emerged as significant in all three populations, with corrected *P*-values of 0.0501, 0.0950, and 0.0607 for CHB, CEU, and YRI, respectively. The enrichment analysis further revealed several pathways and disease related gene sets, including infectious diseases, macroautophagy, interferon signaling, inflammatory biomarkers, B cell receptor (BCR) signaling, and antigen processing and presentation. These findings suggest potential adaptations to local pathogenic environments.

**Table 1.** KOBAS pathway and disease enrichment analysis of population-specific soft sweep genes

Term	#Input	#Background	Corrected *P*-value
**CHB**			
Immune system	37	2,096	3.50E−04
Cytokine signaling in immune system	21	836	5.25E−04
Infectious disease	13	372	1.46E−03
Macroautophagy	7	89	1.48E−03
HIV infection	10	227	1.95E−03
Interferon signaling	9	194	0.0027
Autophagy	7	108	0.0027
Asthma	5	44	0.0033
Phosphorylation of CD3 and TCR zeta chains	4	21	0.0034
Export of viral ribonucleoproteins from nucleus	4	30	0.0071
Hypothyroidism	4	34	0.0083
Metabolism	30	2,075	0.0130
MHC class II antigen presentation	6	123	0.0135
Interleukin-1 family signaling	6	138	0.0193
Interleukin-37 signaling	3	21	0.0197
Autoimmune thyroid disease	4	53	0.0219
Chronic hepatitis B infection	2	5	0.0272
Regulation of glucokinase by glucokinase regulatory protein	3	28	0.0326
Costimulation by the CD28 family	4	65	0.0347
Toxoplasmosis	5	113	0.0361
ISG15 antiviral mechanism	4	69	0.0382
Interactions of Rev with host cellular proteins	3	33	0.0404
Interactions of Vpr with host cellular proteins	3	33	0.0404
Signaling by interleukins	12	619	0.0418
Antiviral mechanism by IFN-stimulated genes	4	77	0.0465
Antigen processing and presentation	4	77	0.0465
NS1 mediated effects on host pathways	3	37	0.0484
Metabolism of lipids	13	728	0.0501
Host interactions with influenza factors	3	39	0.0526
Viral messenger RNA synthesis	3	40	0.0545
Adaptive immune system	13	748	0.0559
Measles	5	138	0.0559
Rheumatoid arthritis	5	144	0.0606
Crohn’s disease	6	220	0.0731
Vitiligo	3	54	0.0862
**CEU**			
Thyroid hormone signaling pathway	6	119	0.0332
HIV-1 control	3	19	0.0356
Triglycerides	5	81	0.0356
Systemic lupus erythematosus and systemic sclerosis	3	21	0.0415
Glaucoma	2	5	0.0475
Inflammatory biomarkers	3	24	0.0475
Sleep duration	2	6	0.0560
Diabetes	4	67	0.0727
Metabolism of lipids	14	728	0.0950
**YRI**			
Obesity-related traits	30	691	2.57E−04
Immune system	59	2,096	0.0020
Adaptive immune system	28	748	0.0050
Signaling by the B cell receptor (BCR)	9	110	0.0129
Antigen processing and presentation	7	77	0.0330
Natural killer cell mediated cytotoxicity	9	131	0.0330
Innate immune system	31	1,043	0.0389
Beta thalassemia/hemoglobin E disease	3	8	0.0418
Congenital disorders of lipid/glycolipid metabolism	8	115	0.0441
Congenital disorders of metabolism	23	695	0.0460
Metabolism of lipids	23	728	0.0607
Metabolism	50	2,075	0.0607
Fatty acid elongation	4	27	0.0607
Downstream signaling events of B cell receptor (BCR)	6	82	0.0920

Significant terms (corrected *P*-value < 0.1) which may be involved in local adaptation are shown on the table.

Moreover, we present the top 10 genes displaying population-specific soft selective sweeps in each population (refer to Table 2, the complete lists of genes under population-specific soft selective sweeps are in supplementary tables S12–S14, Supplementary Material online). These genes are classified into categories based on their functional significance. The first category comprises genes involved in immune response functions, including genes like *AMBRA1*, *PTPRA*, *HLA-DQB1*, and *HRNR* in CHB, *DYNC2H1* in CEU, and *HLA-DQB2*, *HLA-A*, *BGLT3*, *HBBP1*, *HBG1*, and *HBG2* in YRI. By simulations of balancing selection scenarios with selection onset times at 200, 2,000, and 4,000 generations ago, we illustrate that very recent balancing selection may be incorrectly identified as selective sweeps by HaploSweep (supplementary fig. S21, Supplementary Material online). In the initial phases of balancing selection when it is far from equilibrium, the allele frequency trajectory and haplotype pattern resemble those of a selective sweep, owing to the crucial role of heterozygotes in both scenarios. Therefore, early stage balancing selection is difficult to distinguish from selective sweeps, suggesting that the identified HLA genes mentioned above could potentially undergo recent balancing selection.

**Table 2.** The top 10 genes under population-specific soft sweeps in the 1KGP populations CHB, CEU, and YRI

Locus	Position	*P*-value	Description
**CHB**			
*MIR99AHG*	21q21.1	3.13E−13	Obesity-related traits
*AMBRA1*	11p11.2	4.91E−13	Involved in autophagy pathway, Associated with glucose measurement
*RNF38*	9p13.2	4.77E−11	Functional ubiquitin protein ligase
*ATP2C1*	3q22.1	2.22E−10	Contributing to keratinocyte differentiation and epidermis integrity
*WDR74*	11q12.3	3.00E−09	Associated with fatty acid measurement
*FAM227B*	15q21.2	3.98E−09	Associated with goiter
*PTPRA*	20p13	4.41E−09	Involved in signaling by interleukins
*IQCD*	12q24.13	5.10E−09	Associated with body mass index
*HLA-DQB1*	6p21.32	8.17E−09	Major histocompatibility complex, class II
*HRNR*	1q21.3	1.69E−08	Hornerin gene, involved in establishment of skin barrier, involved in innate immune system, associated with hair shape measurement
**CEU**			
*H2AZ1*	4q23	1.96E−11	Involved in metabolism of proteins
*SUPT6H*	17q11.2	2.31E−11	Enables histone binding activity
*KATNAL2*	18q21.1	3.34E−11	Associated with diet measurement
*RSF1*	11q14.1	9.51E−11	Associated with triglyceride measurement
*CBFA2T2, DYNLRB1*	20q11.21-q11.22	2.11E-10	Associated with suntan
*SCAF8*	6q25.2	1.90E−09	Associated with creatinine measurement
*FAM71F2*	7q32.1	8.30E−09	Associated with type 2 diabetes mellitus
*DYNC2H1*	11q22.3	8.85E−09	Involved in Salmonella infection
*TTLL5*	14q24.3	9.68E−09	Related to eye disease
*RSPRY1*	16q13	9.95E−09	Associated with high density lipoprotein cholesterol measurement
**YRI**			
*AK9*	6q21	2.22E−28	Metabolism
*HLA-DQB2, MIR3135B, HLA-A*	6p21.32	1.77E−22	Major Histocompatibility Complex, class II
*SVIL-AS1*	10p11.23	2.13E−20	Associated with body height
*RALGPS1*	9q33.3	5.55E−20	Body height, Alcohol consumption measurement
*ABCG8*	2p21	8.57E−19	Congenital disorders of lipid/glycolipid metabolism
*DDX1*	2p24.3	1.10E−18	Metabolism of proteins
*BGLT3, HBBP1, HBG1, HBG2*	11p15.4	2.01E−18	Malaria resistance
*LPA*	6q25.3-q26	9.32E−16	Involved in cholesterol metabolism
*FMC1-LUC7L2, FMC1*	7q34	1.07E−14	Associated with high density lipoprotein cholesterol
*RANBP2*	2q13	2.41E−14	Associated with hair shape measurement

Only genes with more than five significant SNPs ($P\text{-value}<10^{-4}$) are retained.

The second category encompasses genes associated with metabolic processes, including genes like *AMBRA1*, *WDR74*, and *IQCD* in CHB; *H2AZ1*, *KATNAL2*, *RSF1*, *FAM71F2*, and *RSPRY1* in CEU; and *AK9*, *RALGPS1*, *ABCG8*, *DDX1*, *LPA*, and *FMC1* in YRI. The third category pertains to genes related to phenotypic traits such as skin, hair, or eye phenotypes, including genes like *HRNR* in CHB; *CBFA2T2*, and *TTLL5* in CEU; and *RANBP2* in YRI. These findings suggest that these genes may have undergone selective sweeps driven by various factors including social, environmental, and potentially sexual selection affecting physical characteristics.

### Adaptive Responses to Environmental Factors

Human skin serves as the primary protective barrier against environmental stressors such as toxins, solar radiation and pathogens, plays a critical role in maintaining homeostasis and defense mechanisms (Rees et al. 2020). Hornerin (*HRNR*), a key component in the epidermal cornified cell envelopes, is essential for establishing and maintaining the skin barrier integrity (Henry et al. 2011). *HRNR* expression is up-regulated after experimental disruption of the epidermal barrier (De Koning et al. 2012). A notable soft sweep signal around the *HRNR* gene in the CHB population ($P\text{-value}=1.69\times 10^{-8}$, Table 2) suggests a potential role in local adaptations. This gene was also identified by iHS and nSL with weaker selective sweep signals (iHS P-value=0.0009, nSL P-value=0.0034). Notably, *HRNR* is involved in keratinization, which is the process through which skin cells become tough and impermeable, providing protection against various environmental stressors. Thus, the selective sweep around the *HRNR* gene in the CHB population could represent adaptations to specific environmental factors affecting skin health and protection in this population. Previous research by Eaaswarkhanth et al. (2016) also demonstrated that Asia-specific adaptive forces acting on standing the genetic variation of *HRNR*. The adaptive haplogroup exhibits high frequencies in Asian populations and can be traced back to Africa before the out-of-Africa migrations, as evidenced by its intermediate frequency in present-day Africans (3–16%). Moreover, this haplogroup is present in various ancient Eurasian genomes, including the 45,000-year-old Ust—Ishim genome. These results underscore the efficacy of our method in detecting and distinguishing soft sweep signals.

Another gene, *CBFA2T2*, associated with a reduced tan response to sun exposure as revealed by a genome-wide association study involving 176,678 Europeans (Visconti et al. 2018), is identified as a top signal of soft sweeps in CEU (HaploSweep $P\text{-value}=2.11\times 10^{-10}$, iHS P-value=0.0109 and the nSL P-value=0.0032). Some well-known pigmentation genes under selection in Europeans, such as SLC45A2 and SLC24A5, are absent in the top signals, which may be due to their fixation or near-fixation of functional mutations in Europeans, highlighting the limitations of single-population haplotype-based methods in detecting near-fixation sweeps.

*TTLL5* exhibits a significant soft sweep signal in CEU (HaploSweep $P\text{-value}=9.68\times 10^{-9}$, iHS P-value=0.0117, and nSL P-value=0.0050). This gene is involved in polyglutamylation of *α*-tubulin and is associated with photoreceptor degeneration and solar radiation response, suggests potential adaptations in sensory perception or cellular responses to environmental stimuli in the CEU population (Sergouniotis et al. 2014). Furthermore, *TTLL5* demonstrated a high degree of upregulation in DNA microarray analysis after near-infrared irradiation simulating solar radiation (Tanaka and Nakayama 2016).

Finally, *RANBP2*, associated with straight or curly hair morphology (Adhikari et al. 2016; Endo et al. 2018), may indicate selective pressures on hair-related traits in the YRI population (HaploSweep $P\text{-value}=2.41\times 10^{-14}$, iHS $P\text{-value}=8.79\times 10^{-5}$, and nSL P-value=0.0283). These findings underscore the potential impact of soft sweeps on shaping phenotypic diversity in humans as adaptive responses to environmental pressures.

### Adaptation to Pathogen Resistance

Pathogens play a pivotal role in driving local adaptation within human populations (Fumagalli et al. 2011). In our study, we have identified several genes undergoing strong soft selective sweeps indicative of potential adaptations to distinct pathogenic environments. The gene *HRNR*, previously mentioned, is also involved in the innate immune system and plays a crucial role in defence against pathogens.

Another gene of interest is *DYNC2H1* in CEU (HaploSweep $P\text{-value}=8.85\times 10^{-9}$, iHS $P\text{-value}=7.05\times 10^{-5}$, and nSL P-value=0.0074). This gene is associated with the Salmonella infection pathway. Salmonella infections encompass a spectrum from mild gastroenteritis to severe conditions like typhoid fever and bacteremia. The transition to an agricultural and pastoralist lifestyle during the Neolithic period catalyzed the emergence of human-adapted pathogens. Notably, *Salmonella enterica* has exerted significant pathogenic selective pressure on humans for a long time. Ancient DNA analyses of microbiomes by Key et al. (2020) revealed an increasing specialization towards human hosts from approximately 6.5 thousand years ago, coinciding with transformative cultural shifts among human populations during the agricultural revolution in Western Eurasia, leading to increased exposure to zoonotic diseases like *S. enterica*.

In YRI, a robust soft sweep signal is evident at position 11p15.4, encompassing genes such as *BGLT3*, *HBBP1*, *HBG1*, and *HBG2* (HaploSweep $P\text{-value}=2.01\times 10^{-18}$). The prominent signal occurs 24 kb upstream of *HBB*, a well-known gene associated with malaria resistance (Rockett et al. 2014).

Furthermore, several genes located in the human leukocyte antigen (HLA) region display population-specific soft sweeps, including *HLA-DQB1* (CHB), *HLA-DQB2* (YRI), *MIR3135B* (YRI), and *HLA-A* (YRI). Interestingly, we have identified three HLA genes, namely *HLA-DRB1*, *HLA-DRB5*, and *HLA-DRB6*, undergoing hard sweeps across all three populations. Additionally, the Killer cell immunoglobulin-like receptor (KIR) cluster in 19q13.42 is also experiencing a hard sweep in all three populations. These KIR proteins play a critical role in immune response regulation, with their ligands being specific subsets of HLA class I molecules.

### Adaptation to Diet Style

The influence of agricultural practices on adaptive responses is widespread, with lactase persistence emerging as a prominent example of local adaptation associated with the advent of dairy farming (Ségurel and Bon 2017). In our study, *LCT* has been identified by HaploSweep as undergoing a hard sweep in the CEU population, with the leading SNP exhibiting an extremely significant *P*-value of $1.26\times 10^{-17}$, aligned with former studies. We have identified several novel genes that have undergone soft selective sweeps and may be involved in diet adaptation. *AMBRA1* in CHB (HaploSweep $P\text{-value}=4.91\times 10^{-13}$, iHS and nSL P-value>0.05) is involved in the autophagy pathway. Ambra1 modulates autophagy by regulating the stability and kinase activity of *ULK1* through interaction with *TRAF6* (Kim and Lee 2014). Autophagy plays a pivotal role in regulating body lipid accumulation by controlling adipocyte differentiation and determining the balance between white and brown fat (Singh et al. 2009). Moreover, it maintains energy balance during nutrient deficiency by degrading energy stores, such as proteins, lipid droplets, and glycogen (Kim and Lee 2014). Additionally, autophagy serves as an antibacterial mechanism (Huang and Brumell 2014), suggesting a potential link of the gene *AMBRA1* to pathogenic adaptation. Furthermore, seven genes from the top signals of the soft sweeps are associated with cholesterol or triglyceride measurement, including *AMBRA1*, *WDR74* (in CHB, $P\text{-value}=3.00\times 10^{-9}$), *RSF1* (in CEU, $P\text{-value}=9.51\times 10^{-11}$), *RSPRY1* (in CEU, P-value=9.95$\times 10^{-9}$), *ABCG8* (in YRI, $P\text{-value}=8.57\times 10^{-19}$), *LPA* (in YRI, $P\text{-value}=9.32\times 10^{-16}$), and *FMC1* (in YRI, P-value=$1.07\times 10^{-14}$).

## Discussion

We present a novel method, HaploSweep, to detect and distinguish incomplete soft and hard selective sweeps through haplotype structure. Utilizing the unique haplotype signatures inherent to soft sweeps as a data source, HaploSweep effectively discriminates between these two types of sweeps with improved efficiency. Through extensive simulations that incorporate varying demographic models, selection intensities, degrees of softness, and selection onset times, we demonstrate that HaploSweep outperforms existing popular methods in detecting soft sweeps. This performance gain is markedly noticeable in nonequilibrium populations, such as those experiencing exponential growth. Similar to other single-population haplotype-based methods, including iHS and nSL, HaploSweep has enhanced efficacy in detecting sweep signals with intermediate beneficial allele frequencies. For complete or near-complete sweeps, potential enhancements to the current approach can be achieved by incorporating genomic data from two populations, as demonstrated by methods such as XP-EHH (Sabeti et al. 2007) and XP-nSL (Szpiech et al. 2021).

HaploSweep exhibits high accuracy in distinguishing between soft and hard sweeps, as reflected in the classification accuracies exhibited in CHB, CEU and YRI population simulations based on the human Out-of-Africa model: 0.9247, 0.9484, and 0.9829, respectively. In particular, the classification accuracy is relatively robust in different demographic models used in the simulation. However, it should be noted that the effectiveness of classifying sweep types is influenced by population bottlenecks, which can result in a decreased degree of softness for soft sweeps. Consequently, the classification accuracy is superior for the YRI populations compared to the CHB and CEU populations.

A notable challenge of detecting soft sweeps lies in the identification of a soft shoulder, where hard sweeps may display signatures resembling those of soft sweeps and partial sweeps at nearby loci. Simulation demonstrates that the flank region of a recent strong hard sweep is prone to being recognized as a soft sweep by HaploSweep. However, the leading significant SNPs in the same region are accurately identified as part of the hard sweep. To address this challenge, we delineate a strategy to precisely demarcate the sweep-affected zones influenced by a soft shoulder. Simulation results demonstrate the efficacy of this strategy. Another challenge is to infer the essential parameters of the soft selective sweep process, such as selection intensity and selection onset time, which can only be achieved through explicit modeling of the haplotype structure (Chen et al. 2015).

HaploSweep has several limitations. Unlike H-statistics, HaploSweep requires the polarization of alleles as ancestral or derived, along with known genetic positions of the SNPs. In contrast, the nSL statistic does not need genetic position information. HaploSweep is designed for phased haplotype data, while G-statistics and diploS/HIC accommodate unphased genotypes. Compared to iHS, H12, and nSL, HaploSweep requires a larger sample size to achieve sufficient power in detecting and classifying sweeps. With sample sizes below approximately ∼50 haplotypes, capturing the sub-haplotype structure caused by distinct founder haplotypes becomes challenging due to insufficient sample sizes for each cluster. HaploSweep, iHS, and nSL are designed to detect only partial sweeps, whereas H-statistics and ML methods are capable of detecting both partial and complete sweeps. Additionally, HaploSweep cannot distinguish early-stage balancing selections (not yet at equilibrium) from selective sweeps.

In the genome-wide scan for recent selective sweeps of three populations (CHB, CEU, and YRI) from the 1,000 Genomes Project, we identify numerous genes associated with immune response, metabolic functions, and appearance phenotypes in the top list of population-specific soft selected genes. A notable example is Hornerin (*HRNR*), a constituent of epidermal cornified cell envelopes crucial for the establishment of the skin barrier. Previous studies have implicated *HRNR* under a soft selective sweep in the CHB population, supported by haplogroup frequency distributions in contemporary and ancient populations. HaploSweep analysis also revealed multiple genes undergoing strong population-specific soft sweeps that were previously unreported. These novel findings contribute to our understanding of the adaptive evolution of populations in response to their local natural environments, pathogens, and dietary patterns. Enrichment analysis further highlighted terms related to immune response and metabolism, such as metabolism of lipids, infectious disease, macroautophagy, and interferon signaling, underscoring the rapid adaptive evolution of human populations in diverse environments.

## Materials and Methods

### HaploSweep Statistics iHSL and RiHS

HaploSweep consists of two statistics, namely iHSL and RiHS. HaploSweep requires phased data and information on the ancestral/derived allele at each segregating site. Suppose the input data contains a total of *n* haplotypes, represented as $H=\{h_{1},h_{2},\ldots ,h_{n}\}$. Each haplotype $h_{j}$ is a vector of *m* elements, with the entry *j* encoded as 0 or 1 to indicate the allele type of the *i*-th SNP on the *j*th chromosome. The physical and genetic positions of the SNPs are also assumed known. Denote C as the set of all possible distinct haplotypes at a locus of interest ($x_{0}$). A local haplotype $h_{j}(x_{0})$ represents a segment of $h_{j}$ centered around the locus of interest ($x_{0}$), including *L* SNP both upstream and downstream.

If $x_{0}$ is undergoing a soft selective sweep, $H(x_{0})=\{h_{1}(x_{0}),\ldots ,h_{n}(x_{0})\}$ can be partitioned into nonoverlapping clusters that can be traced back to distinct ancestral haplotypes carrying the beneficial allele. These local haplotypes experiencing a soft sweep typically form closely interconnected cluster within sub-lineages originating from a single ancestral haplotype, along with noticeable separation among different sub-lineages. The haplotype homozygosity decreases within the clusters. However, in practice, distinguishing between different clusters and solving the bias in calculating haplotype homozygosity can be challenging. To address this, we propose a definition for clusters: for each haplotype $h_{j}$, the local cluster around $x_{0}$ is a subset of *H* defined as,


(1) $$H_{j}=\{h_{j},h_{j}^{1},h_{j}^{2},\ldots ,h_{j}^{s}\},$$

with $h_{j}^{s}(x_{0})\in H(x_{0})$ represents the top *s* local haplotypes most similar to $h_{j}(x_{0})$ by Hanming distance. *s* is defined as the diameter of the local cluster $H_{j}$.

Let $H(x_{i},h_{j})$ be a partition of $C(x_{i})$ containing all distinct haplotypes in the local cluster $H_{j}$. We calculate the EHH of the chromosomes in local cluster $H_{j}$ to marker $x_{i}$ as


(2) $$\text{EHH}\left(x_{i},h_{j}\right)=\sum_{h\in H\left(x_{i},h_{j}\right)}\frac{\left(\begin{array}{c} n_{h}\\ 2 \end{array}\right)}{\left(\begin{array}{c} \left|H_{j}\right|\\ 2 \end{array}\right)},$$

where $n_{h}$ is the number of observed haplotypes of type $h\in H(x_{i},h_{j})$ and $\left|H_{j}\right|$ is the number of haplotypes in local cluster $H_{j}$.

The integrated haplotype homozygosity for local cluster $H_{j}$ ($\text{iHH}(h_{j})$) is calculated using trapezoidal quadrature:


(3) $$\begin{array}{rl} \text{iHH}\left(h_{j}\right) & =\sum_{i=1}^{\left|D\right|}\frac{1}{2}\left(\text{EHH}\left(x_{i-1},h_{j}\right)+\text{EHH}\left(x_{i},h_{j}\right)\right)g\left(x_{i-1},x_{i}\right)\\ & \;+\sum_{i=1}^{\left|U\right|}\frac{1}{2}\left(\text{EHH}\left(x_{i-1},h_{j}\right)+\text{EHH}\left(x_{i},h_{j}\right)\right)g\left(x_{i-1},x_{i}\right). \end{array}$$

Here, D is the set of markers downstream from the current locus, such that $x_{i}\in D$ denotes the *i*-th closest downstream marker from the locus of interest ($x_{0}$). U and $x_{i}\in U$ are defined similarly for upstream markers. $g(x_{i-1},x_{i})$ is the genetic distance between two markers.

The integrated haplotype homozygosity for the ancestral (0) and derived (1) haplotypes (${\text{iHH}}_{c}$, *c* is either 0 or 1) is defined as the mean of $\text{iHH}(h_{j})$ for haplotype $h_{j}$ carrying *c* on the target marker $x_{0}$,


(4) $${\text{iHHL}}_{c}=\frac{1}{\left|H_{c}\right|}\sum_{h_{j}\in H_{c}}\text{iHH}\left(h_{j}\right),$$

where $H_{c}$ denotes all haplotypes that carry *c* on the marker $x_{0}$. The unstandardized iHSL (uiHSL) is then calculated as:


(5) $$\text{uiHSL}=\text{ln}\left(\frac{{\text{iHHL}}_{1}}{{\text{iHHL}}_{0}}\right).$$

Low frequency alleles are generally younger and are associated with longer haplotypes than higher frequency alleles. Considering the bias of allele age, the unstandardized scores are normalized in frequency bins across the entire genome (Sabeti et al. 2007),


(6) $$\text{iHSL}=\frac{\text{ln}\left(\frac{{\text{iHHL}}_{1}}{{\text{iHHL}}_{0}}\right)-E_{p}\left[\text{ln}\left(\frac{{\text{iHHL}}_{1}}{{\text{iHHL}}_{0}}\right)\right]}{SD_{p}\left[\text{ln}\left(\frac{{\text{iHHL}}_{1}}{{\text{iHHL}}_{0}}\right)\right]},$$

where $E_{p}[\text{ln}(\frac{{\text{iHHL}}_{1}}{{\text{iHHL}}_{0}})]$ and $SD_{p}[\text{ln}(\frac{{\text{iHHL}}_{1}}{{\text{iHHL}}_{0}})]$ are the expectation and standard deviation in frequency bin *P*.

We set the diameter of the local cluster to $s=\min(\max(r|H_{c}|,8),|H_{c}|)$, where r=0.1, and the length of the local haplotype as L=400. The statistic iHS is a special case of iHSL when set r=1. The statistic iHSL is expected to gain more power on detecting soft sweep when setting a smaller value of *r* in large samples.

The unstandardized statistic RiHS is defined as the logarithmic ratio between iHHL and iHH,


(7) $${\text{uRiHS}}_{c}=\text{ln}\left(\frac{{\text{iHHL}}_{c}}{{\text{iHH}}_{c}}\right).$$

The RiHS statistic is obtained by further standardizing the unstandardized RiHS,


(8) $${\text{RiHS}}_{c}=\frac{\text{ln}\left(\frac{{\text{iHHL}}_{c}}{{\text{iHH}}_{c}}\right)-E_{p}\left[\text{ln}\left(\frac{{\text{iHHL}}_{c}}{{\text{iHH}}_{c}}\right)\right]}{SD_{p}\left[\text{ln}\left(\frac{{\text{iHHL}}_{c}}{{\text{iHH}}_{c}}\right)\right]},$$

where c=1 if iHSL ≥0, and c=0 if iHSL <0. In the context of a soft sweep, we expect a higher RiHS value for the selected allele compared to neutral evolution and hard sweeps. Thus, the statistic RiHS serves as a valuable tool for detecting selective sweeps and distinguishing between different sweep types.

### Coalescent Simulation

The coalescent simulator msms is used to generate simulation data sets (Ewing and Hermisson 2010). Each segment has a length of 2 Mb. In simulations involving selective sweeps, the advantageous allele is positioned at the center of the segments. The mutation rate is set to $\mu =2.5\times 10^{-8}$ per base pair per generation (Nachman and Crowell 2000; Campbell et al. 2012), and the recombination rate is set to $r=1.25\times 10^{-8}$ per base pair per generation, consistent with observed rates in human populations (Jensen-Seaman et al. 2004). To comprehensively evaluate the performance of HaploSweep in detecting and distinguishing incomplete hard and soft selective sweeps, we simulate data under various demographic models. Additional details are provided below.

#### Equilibrium Demographic Model

We simulate an equilibrium demographic model with a constant effective population size of Ne=10,000 (supplementary fig. S1, Supplementary Material online). In this model, we consider two types of soft selective sweeps: those arising from standing genetic variation and those from recurrent de novo mutations. For soft sweeps from standing genetic variation, we vary the starting frequencies of the beneficial allele ($f_{0}$) at 0.05,0.1,0.2, the selection intensities (*s*) at 0.01,0.02,0.05, and the selection onset time at 100–2,000 generations ago. For each parameter combination, 1,000 simulated data are generated. The simulations are categorized into six sets based on the adaptive allele frequency in the contemporary population sample, i.e. 0.25≤f<0.35, 0.35≤f<0.45,…, 0.75≤f≤0.85. For soft sweeps from recurrent de novo mutations, we set the scaled mutation rate to $\theta =4N_{e}\mu =5,10,50$, the frequency of the beneficial allele in the contemporary population sample at f=0.3,0.4,…,0.8, and the selection intensities at s=0.01,0.02,0.05. Hard sweeps are simulated with parameters identical to those of soft sweeps from recurrent de novo mutations, except for the scaled mutation rate set to θ=0.01. For soft sweep simulations, samples with fewer than 2 founder lineages are excluded, while for hard sweep simulations, samples with more than 1 founder lineages are excluded.

To assess the robustness of HaploSweep under varying recombination and mutation rates, we generate neutral segment simulation datasets with recombination rates ranging from 0.2,0.3,…,0.9 times the normal value, and mutation rates ranging from 1.2,1.4,…,3 times the normal value. The sample size for these datasets is set to 500 individuals.

#### Nonequilibrium Demographic Model

We conduct simulations for three nonequilibrium demographic models: (i) Exponential Growth Model, where the effective population size (*Ne*) increases from 1,000 to 20,000 over 1,000 generations, with a growth rate of 0.3% per generation. (ii) Mild Bottleneck Model, where *Ne* is reduced to half of the normal value starting 300 generations ago and lasting for 50 generations. (iii) Severe Bottleneck Model, where *Ne* is set to 0.1 times the normal value, starting 300 generations ago and lasting for 50 generations. In these nonequilibrium demographic models, we set a fixed selection intensity of s=0.02 and a fixed initial frequency of the adaptive allele at $f_{0}=0.1$. Selection onset times are set to 200, 300, and 400 generations backward in time. Similar to the soft sweep simulations in the equilibrium model, we categorize the simulations into six sets based on the adaptive allele frequencies in the contemporary population samples. The sample size of these datasets is also fixed to 500 individuals.

#### Human Out-of-Africa Demographic Model

We conduct simulations for the CHB, CEU, and YRI populations using the human out-of-Africa demographic model (Gravel et al. 2011). The sample sizes match that of unrelated individuals in the 1KGP dataset, with 103, 99, and 108 unrelated individuals from the three population, respectively. For soft sweeps, the initial allele frequencies of the adaptive allele are generated from a uniform distribution U(0.01,0.2), while selection intensities are generated from U(50,1,000), and selection onset times from U(0,2,000) generations ago. For hard sweeps, the selection intensities and selection onset times follow the same distributions as those for soft sweeps. To improve efficiency, the initial frequencies for hard sweeps are generated from U(0,0.001), and simulation data with multiple founder haplotypes are filtered out. Additionally, simulation data with a single founder haplotype are filtered out for the soft sweeps. Only simulation data with adaptive allele frequencies between 0.05 and 0.95 are retained. A total of 100,000 samples are generated for neutral evolution, soft sweeps, and hard sweeps, respectively. HaploSweep statistics are calculated for the 300,000 simulation datasets, and the simulated distribution of HaploSweep values for CHB, CEU, and YRI is obtained.

### Applying HaploSweep to 1 KGP Populations CHB, CEU, and YRI

A genome-wide scan for selective sweeps is carried out using HaploSweep in three populations: CHB, CEU, and YRI from the high-coverage 1,000 Genomes Project (Byrska-Bishop et al. 2022). Each population consists of 103, 99, and 108 unrelated individuals, respectively. The genetic position for each SNP is determined via linear interpolation from the genetic map provided by Shapeit5 (Hofmeister et al. 2023). The HaploSweep statistics are calculated along the genome. For RiHSL, P-value=F(RiHSL), where F(x) denotes the cumulative distribution function for the chi-square distribution with 2 degrees of freedom. For iHSL, P-value=2Φ(−|iHSL|), where iHSL is the normalized iHSL score and Φ(x) denotes the cumulative distribution function for the standard normal distribution. We identify the 200 HaploSweep data points that are closest to the target SNP in terms of the HaploSweep statistics (RiHS and iHSL) from the simulated distribution under the Human out-of-Africa demographic model. The sweep type of the target SNP is then determined by the majority sweep type among these 200 data points.

### Calculating the Power of HaploSweep on Simulated Data

After normalization, iHSL scores follow a standard normal distribution. The power to detect the adaptive allele in the simulated data (derived allele in our simulations) at a 1% FPR is calculated as the proportion of scores exceeding 2.3263 (one-tailed test). RiHSL scores follow a chi-square distribution with 2 degrees of freedom. Power at a 1% FPR is calculated as the proportion of RiHSL scores exceeding 9.2103, with normalized iHSL scores greater than 0.

### Calculation of iHS and nSL

The statistics iHS and nSL are computed using selscan 2.0 (Szpiech 2024). Default parameter settings are utilized for iHS calculation. For nSL, we specify a maximum extension distance of 500 for an nSL haplotype (–max-extend-nsl 500), while remaining default values for other settings. After normalization, both iHS and nSL scores follow a standard normal distribution. The power to detect the adaptive allele (derived allele in our simulated data) under a 1% FPR is determined by the proportion of scores exceeding 2.3263 in a one-tailed test.

### Calculation of H-statistics

We calculate H12 and H2/H1 using an SNP-based window following Garud et al. (2015). Various window sizes—21, 51, 101, 201, 401, 1,001, and 2,001—were tested to determine the optimal size yielding the highest power. For each simulated segment under selective sweep, the H-statistics are calculated with a window centered on the adaptive site. Similarly, the H-statistics are calculated with a window centered on the middle site for each simulated neutral segment. Power for the H12 statistic (at a 1% FPR) is determined by the proportion of scores exceeding the 99th percentile of the distribution of scores from neutral simulations.

## Supplementary Material

msae192_Supplementary_Data

## Data Availability

The source code for HaploSweep is available on GitHub at https://github.com/ChenHuaLab/HaploSweep. The genomic sequences for CHB, CEU, and YRI were obtained from the 1,000 Genomes Project at https://www.internationalgenome.org.
