# Supplementary material for: HaploSweep: Detecting and Distinguishing Recent Soft and Hard Selective Sweeps through Haplotype Structure
Source: Mol Biol Evol. 2024 Sep 17;41(10):msae192. doi: 10.1093/molbev/msae192 (PMC11452351; doi:10.1093/molbev/msae192)
Supplement: msae192_Supplementary_Data [file msae192_supplementary_data.zip › SupplementaryInformation.pdf]

Supplementary information for: HaploSweep: detecting and distinguishing recent soft and hard selective sweeps through haplotype structure

Shilei Zhao[a,b,c,1], Lianjiang Chi[a,b,c,1], Mincong Fu[a,b,c], Hua Chen[a,b,c,d,*]

[a] *Beijing Institute of Genomics, Chinese Academy of Sciences, Beijing 100101, China*
[b] *China National Center for Bioinformation, Beijing 100101, China*
[c] *School of Future Technology, University of Chinese Academy of Sciences, Beijing 100049, China*
[d] *CAS Center for Excellence in Animal Evolution and Genetics, Chinese Academy of Sciences, Kunming 650223, China*

- Fig. S1 illustrates the simulation settings for different demographic models, with mutation and recombination rates chosen following Vy *et al.* (2017). Parameters of the human out-of-Africa model are adopted from Gravel *et al.* (2011). Simulations are conducted using the MSMS coalescent simulator (Ewing and Hermisson, 2010).

- Fig. S2 presents the power to detect hard sweep signals under varying selection intensities for iHSL, iHS, H12, and nSL. Notably, nSL exhibits superior performance in detecting hard sweep signals.

- Fig. S3 shows the power of H12 with different window sizes on simulations of soft sweeps from standing variation (see the *Equilibrium demographic model* in the *Coalescent simulation* section) with different parameters. Fig. S4 presents the power of H12 with different window sizes on simulations of soft sweeps from recurrent mutations with different parameters. The SNP-based window size that achieved the highest mean power across different parameters is used in subsequent calculation of H-statistics.

- Figs. S5-S7 present EHH curves for iHS and iHSL under a soft selective sweep with different selection intensities.

- Fig. S8 presents the power of HaploSweep, iHS, H12, and nSL in detecting soft selective sweeps from recurrent mutations with selection coefficients of 0.001, 0.002, and 0.005.

- Figs. S9 and S10 illustrate the power of HaploSweep, iHS, H12, and nSL in detecting soft

*Corresponding author: chenh@big.ac.cn
[1] These authors contributed equally.

selective sweeps from recurrent mutations with sample sizes of 100 haplotypes and 50 haplotypes, respectively.

- Fig. S11 shows the performance of HaploSweep in pinpointing the adaptive locus.

- Fig. S12 displays the distribution of RiHSL and the Pearson correlation coefficient between iHSL and RiHS for simulated neutral data and real data from CHB, CEU, and YRI populations.

- Fig. S13 demonstrates the performance of iHSL and RiHSL in detecting soft sweep signals, and RiHSL shows higher power at low allele frequencies but lower power at high allele frequencies.

- Fig. S14 illustrates the false positive rates for iHSL, iHS, H12, and nSL across a range of recombination and mutation rates. H12 is sensitive to variations in these rates.

- Fig. S15 shows the distribution of normalized iHSL values for soft selective sweeps simulated with varying mutation rates. The selection intensity is set to $s = 0.01$, the frequency of the beneficial allele in the contemporary population sample is $f = 0.5$, and the scaled mutation rate of the beneficial allele is $\theta = 10$. The background mutation rates are set to $\mu = 0.5 \times 10^{-8}$, 1×10^{-8}, 1.5×10^{-8}, 2×10^{-8}, and 2.5×10^{-8} per bp per generation.

- Fig. S16 presents the confusion matrices of HaploSweep, H-statistics, and diploS/HIC for simulated data of populations CHB (206 haplotypes), CEU (198 haplotypes), and YRI (216 haplotypes, 100 haplotypes, and 50 haplotypes). DiploS/HIC identifies two additional classes, Hard-Linked and Soft-Linked, compared to HaploSweep and H-statistics. To better compare the three methods, we merge the five classes into three: we remove the true classes of Hard-Linked and Soft-Linked; the predicted class of Hard-Linked is regarded as hard sweep, and the predicted class of Soft-Linked is regarded as soft sweep. After merging, the classification accuracies for HaploSweep, H-statistics, and diploS/HIC are 0.7807, 0.6121, and 0.5733 for CHB (206 haplotypes); 0.7962, 0.6239, and 0.5833 for CEU (198 haplotypes); 0.8209, 0.6738, and 0.6633 for YRI (216 haplotypes); 0.7784, 0.6242, and 0.6267 for YRI (100 haplotypes); and 0.7241, 0.5897, and 0.5867 for YRI (50 haplotypes). Note that these results are all for partial sweeps.

- Fig. S17 assesses the classification of sweep types under incorrectly-specified demographic history. HaploSweep demonstrates robustness under uncertain demographic models.

- Figs. S18-S20 depict the Manhattan plots of HaploSweep, iHS, and nSL for CHB, CEU, and YRI populations, respectively.

- Fig. S21 shows the proportions of balancing selection misclassified as selective sweeps by HaploSweep. Balancing selection is simulated with different onset times: T=200, 2000, and 4000 generations ago. Very recent balancing selections (T=200) with low initial allele frequencies (f_0=0.01) are prone to be misclassified as selective sweeps by HaploSweep. In the early stages of balancing selection (far from equilibrium), the allele frequency trajectory and haplotype extension resemble those of a selective sweep, as heterozygotes play a critical role in both scenarios. Thus, it is difficult to distinguish early-stage balancing selection from selective sweeps.

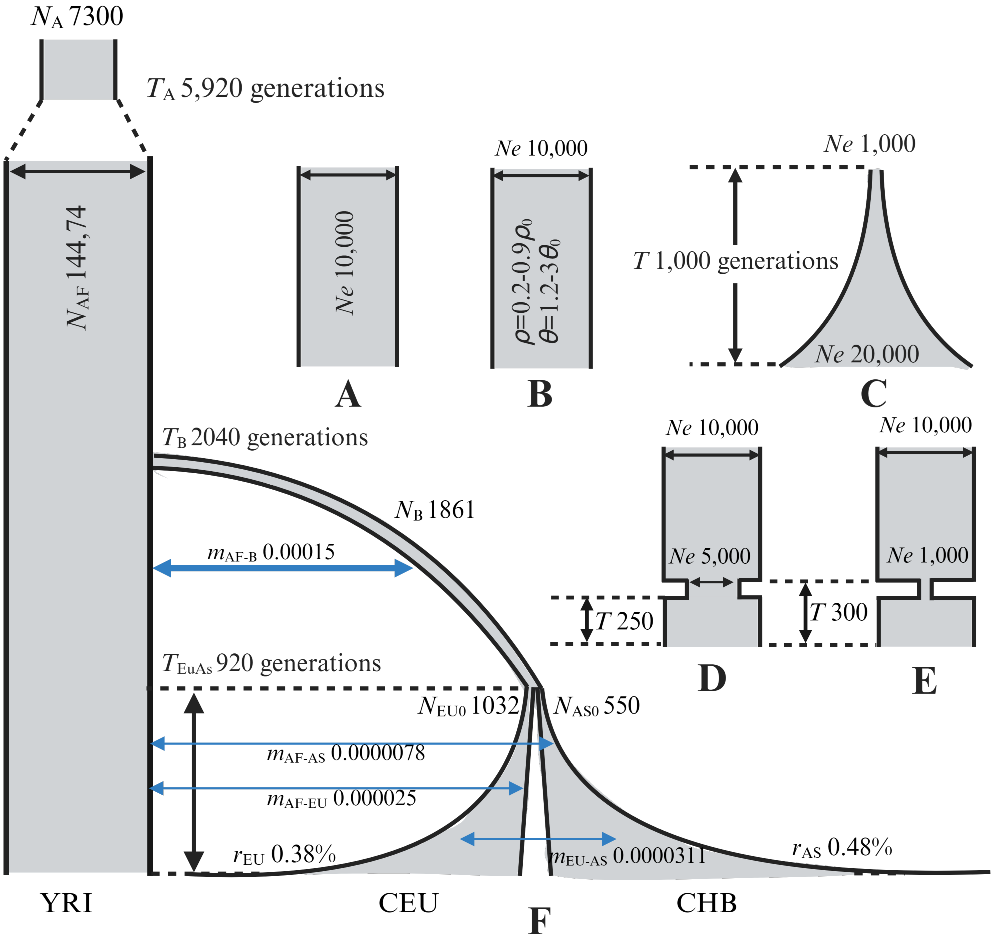

Fig. S1. Simulation setting of different demographic models. A) Equilibrium demographic model with a constant effective population size of $Ne = 10,000$, mutation rate of 2.5×10^{-8} per site per generation and recombination rate of 1.25×10^{-8}. B) Similar to A) but with the recombination rate ranging from $0.2, 0.3, ..., 0.9$ times the normal value and the mutation rate ranging from $1.2, 1.4, ..., 3$ times the normal value. C) Exponential growth model. The population size (Ne) grows from $1,000$ to $20,000$ over $1,000$ generations, with a growth rate of 0.3% per generation. D) Mild Bottleneck Model. Ne is set to half of the normal value, starting from 300 generations ago and lasting for 50 generations. E) Severe Bottleneck Model. Ne is set to 0.1 times the normal value, starting from 300 generations ago and lasting for 50 generations. F) Human out-of-Africa model (Gravel *et al.*, 2011).

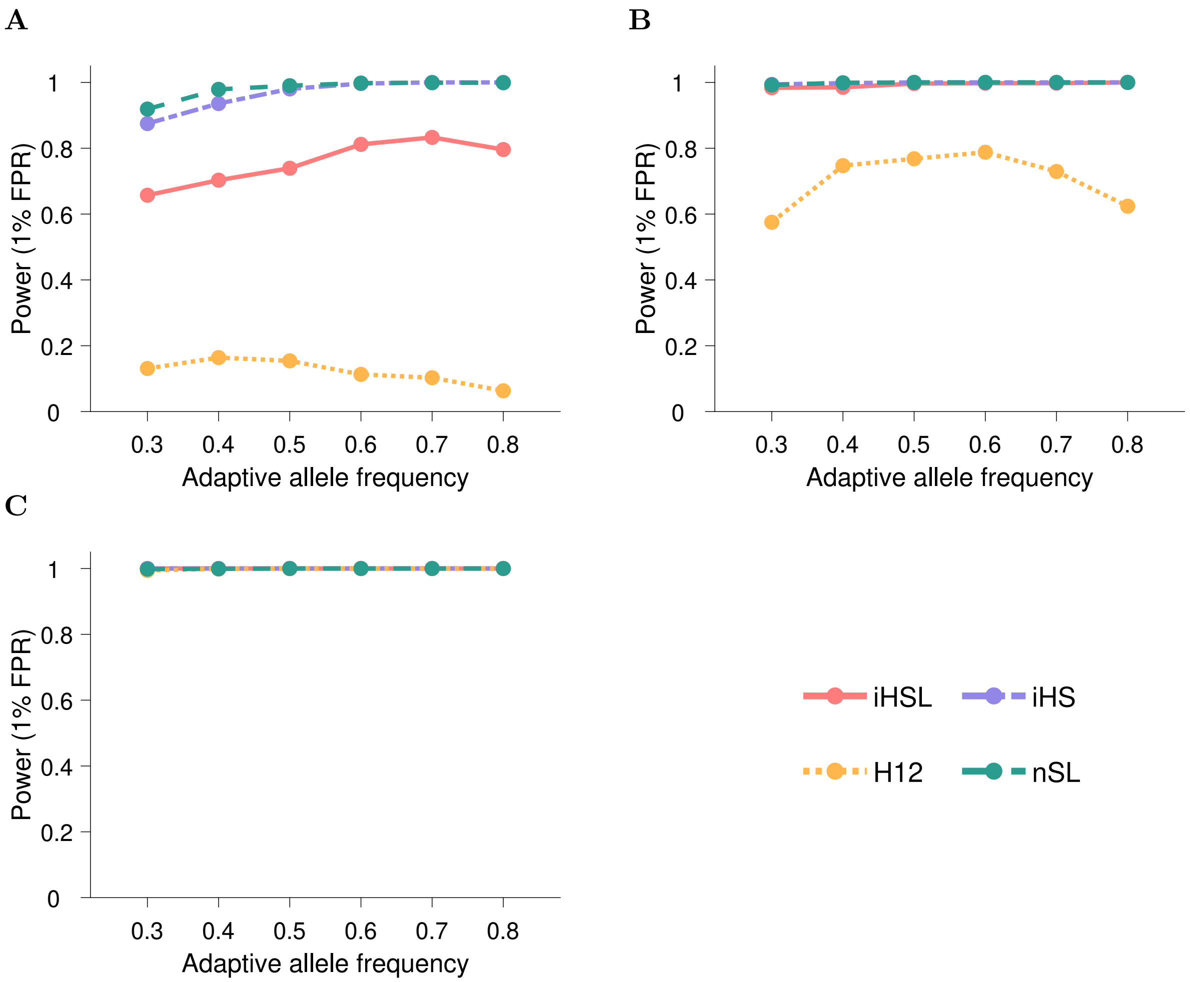

Fig. S2. Comparison of power to detect hard sweep under different selection intensities (s). A) $s = 0.01$, B) $s = 0.02$, and C) $s = 0.05$.

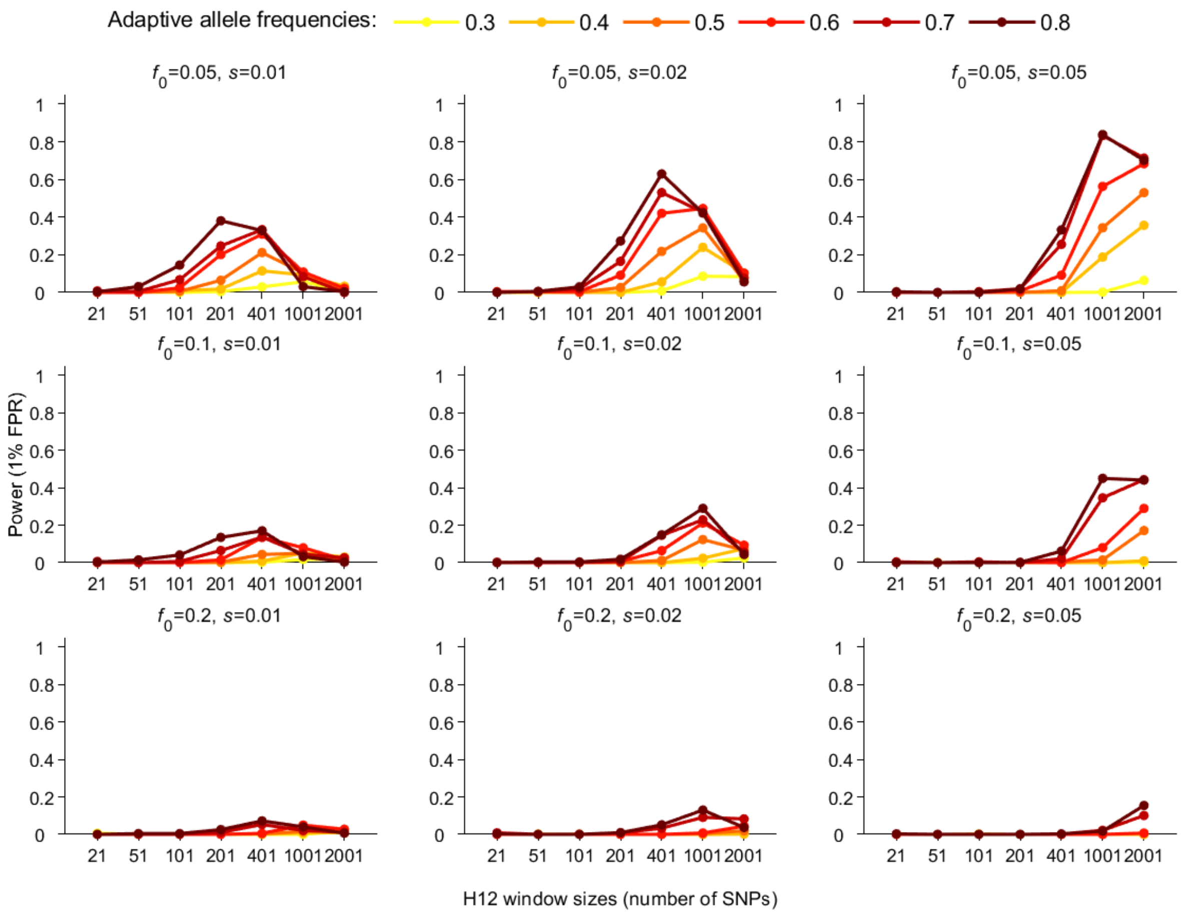

Fig. S3. Power of H12 on simulations of soft sweeps from standing variation with different parameters. The optimal window size is 1001 SNPs, yielding the highest mean power of 0.3049.

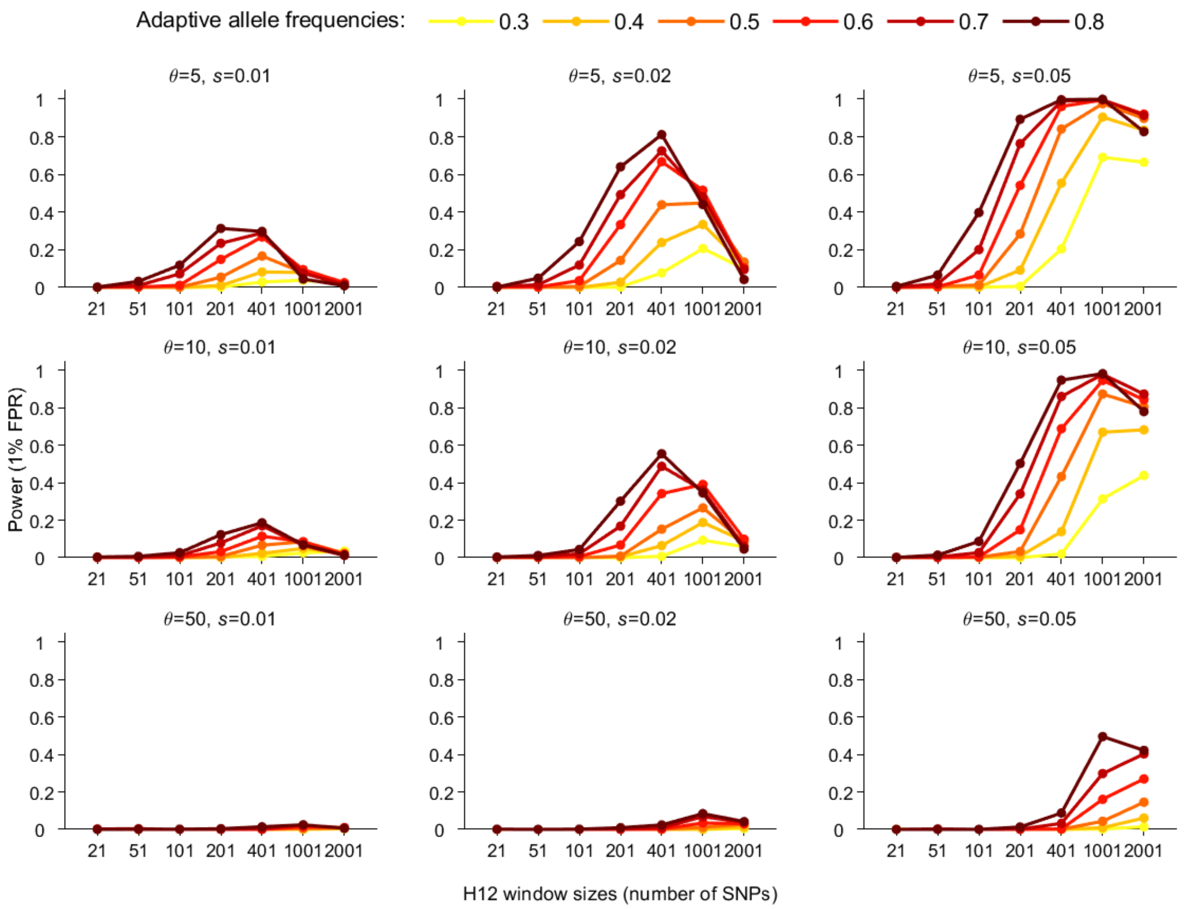

Fig. S4. Power of H12 on simulations of soft sweeps from recurrent mutations with different parameters. The optimal window size is 1001 SNPs, yielding the highest mean power of 0.2233.

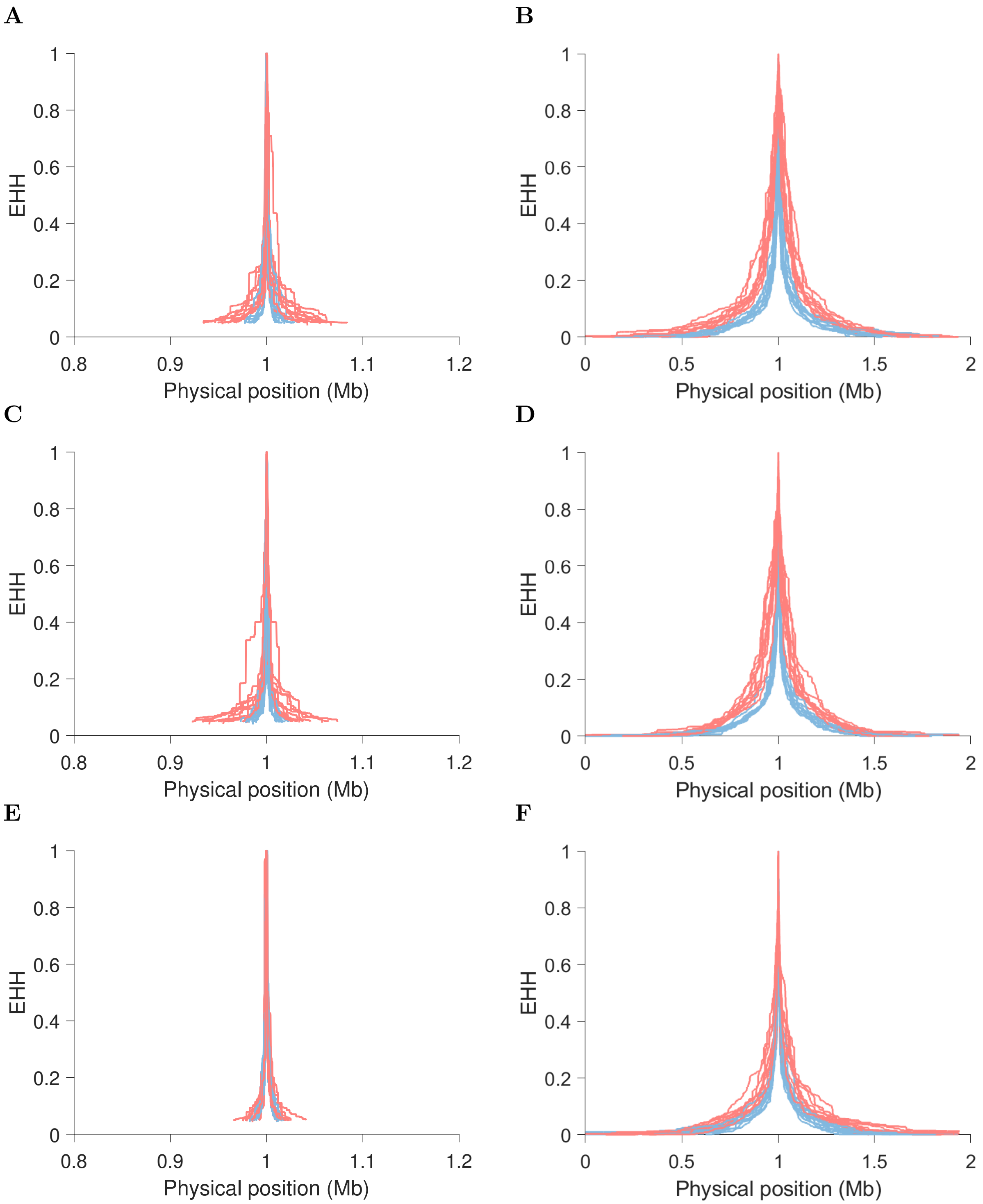

Fig. S5. EHH curves of iHS and iHSL for samples under a soft selective sweep with the selection intensity of $s = 0.01$. The red and blue curves represent the EHH curves for haplotypes carrying the adaptive and neutral alleles across ten simulation replicates. The adaptive allele frequency in the sample of the contemporary population is 0.5. Panels A, C, E show the EHH curves for iHS with the scaled mutation rates $\theta = 5, 10, 50$ respectively. Panels B, D, F show the EHH curves for iHSL with the scaled mutation rates $\theta = 5, 10, 50$ respectively.

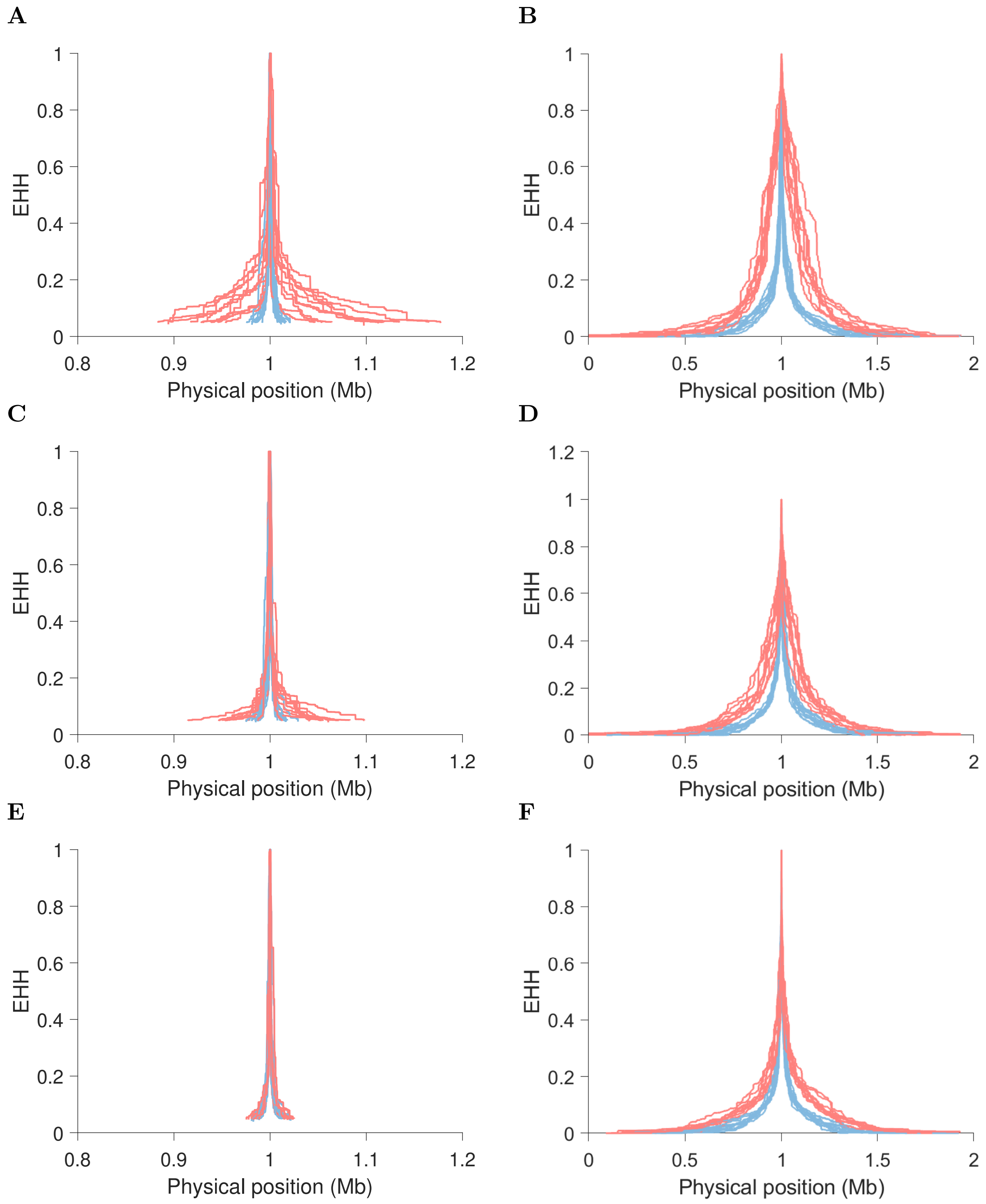

Fig. S6. EHH curves of iHS and iHSL for a sample under a soft selective sweep with a selection intensity of $s = 0.02$. The red and blue curves represent the EHH curves for haplotypes carrying the adaptive and neutral alleles across ten simulation replicates. The adaptive allele frequency in the sample of the contemporary population is 0.5. Panels A, C, E show the EHH curves for iHS with the scaled mutation rates $\theta = 5, 10, 50$ respectively. Panels B, D, F show the EHH curves for iHSL with the scaled mutation rates $\theta = 5, 10, 50$ respectively.

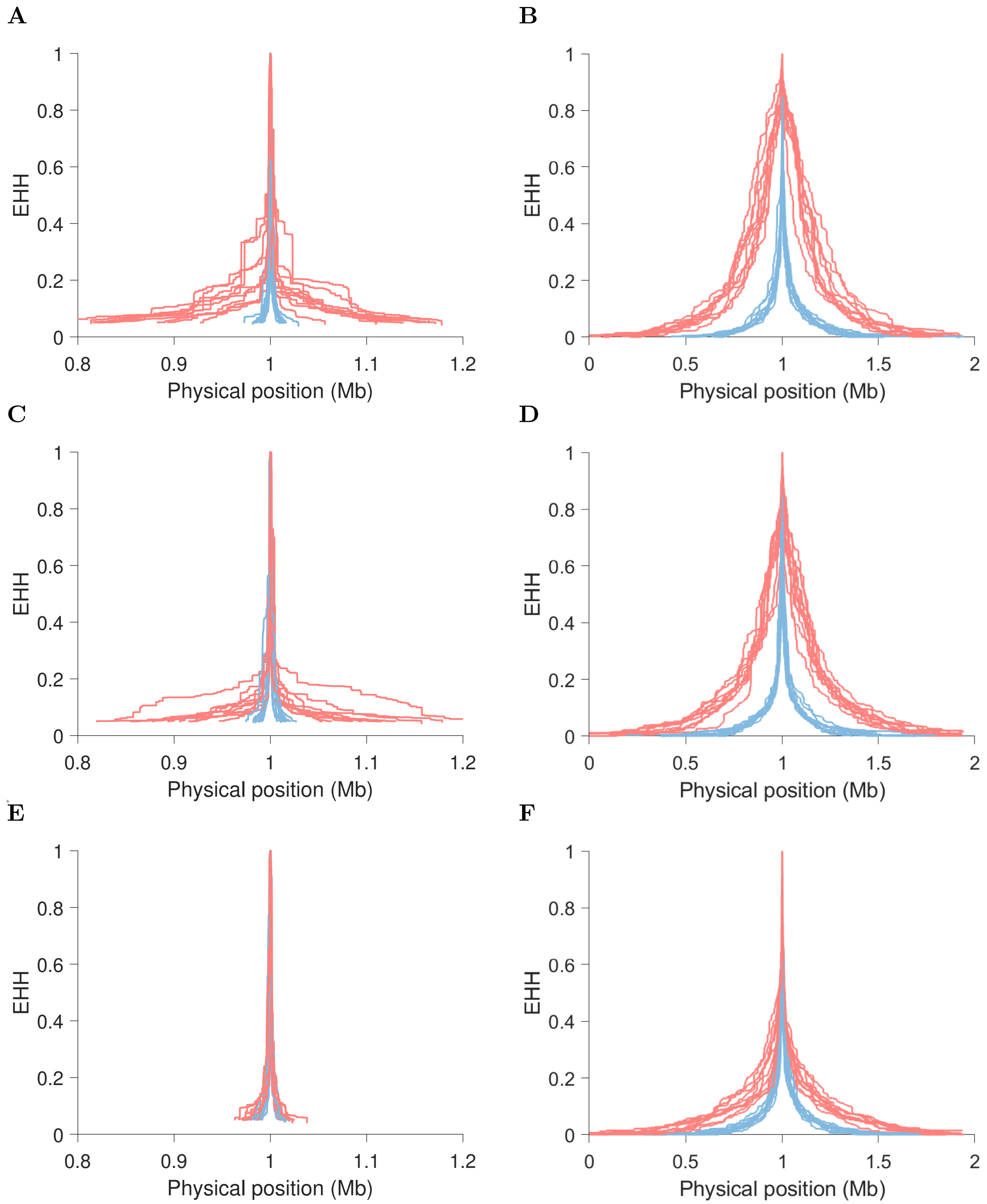

Fig. S7. EHH curve of iHS and iHSL for samples under a soft selective sweep with a selection intensity of $s = 0.05$. The red and blue curves represent the EHH curves for haplotypes carrying the adaptive and neutral alleles across ten simulation replicates. The adaptive allele frequency in the sample of the contemporary population is 0.5. Panels A, C, E show the EHH curves for iHS with the scaled mutation rates $\theta = 5, 10, 50$ respectively. Panels B, D, F show the EHH curves for iHSL with the scaled mutation rates $\theta = 5, 10, 50$ respectively.

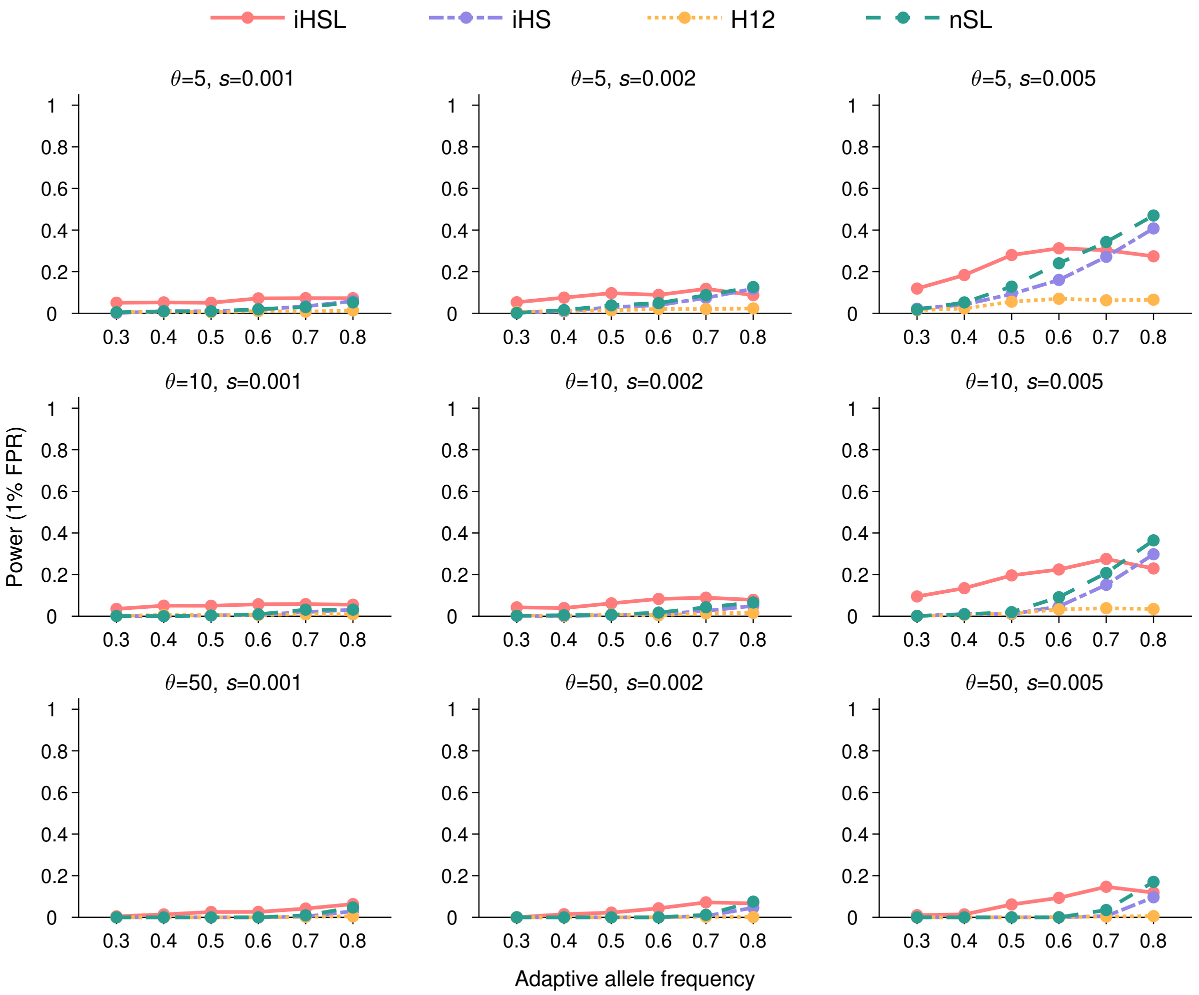

Fig. S8. Comparison of the power of four methods for detecting soft selective sweeps from recurrent mutations across weaker selection coefficients of 0.001, 0.002, and 0.005. The window size for H12 is set to 401 SNPs, which exhibited the highest mean power among seven tested window sizes ranging from 21 to 2001.

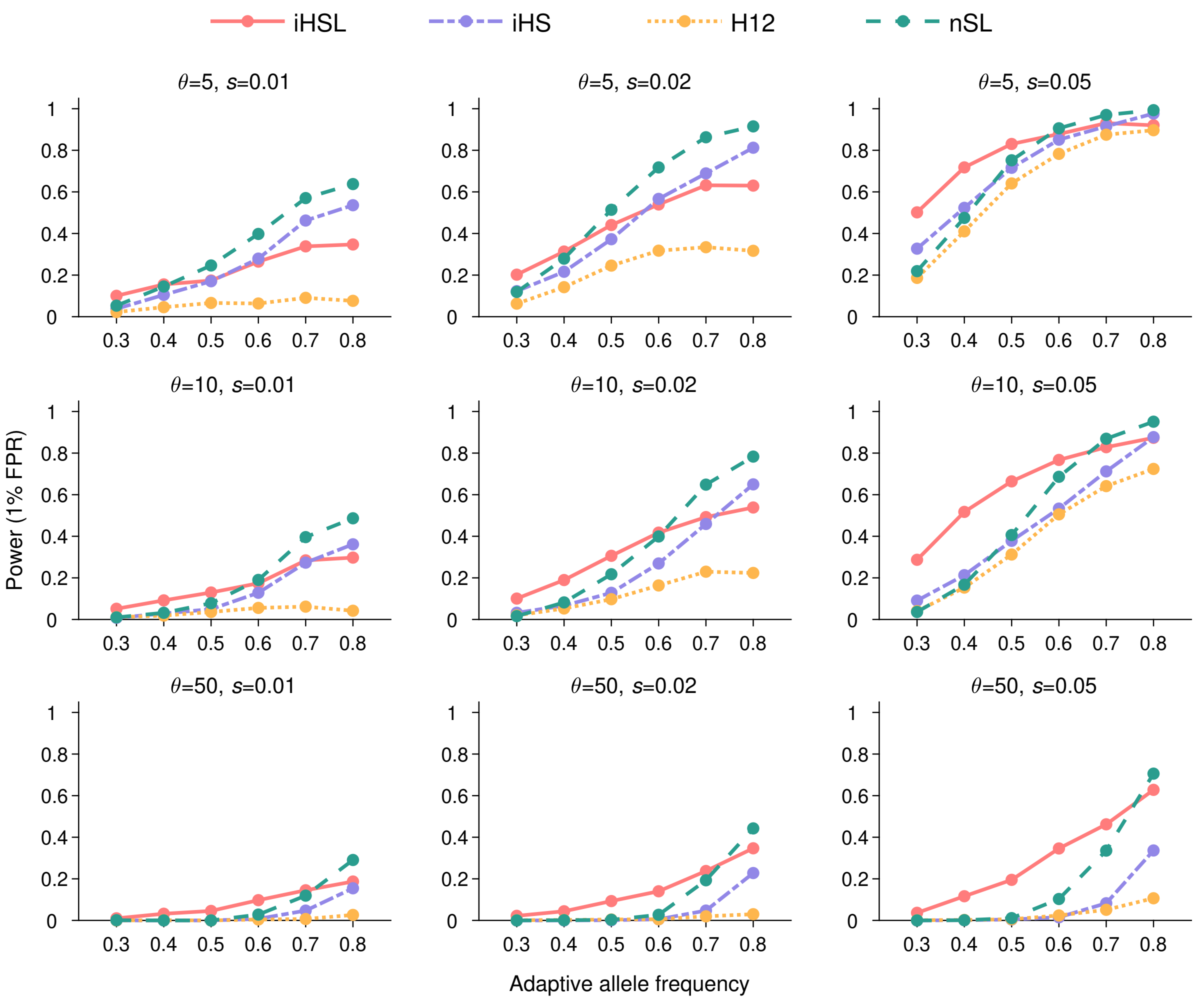

Fig. S9. Comparison of the power of four methods for detecting soft selective sweeps from recurrent mutations with a sample size of 100 haplotypes. The window size for H12 is set to 401 SNPs, which exhibited the highest mean power among seven tested window sizes ranging from 21 to 2001.

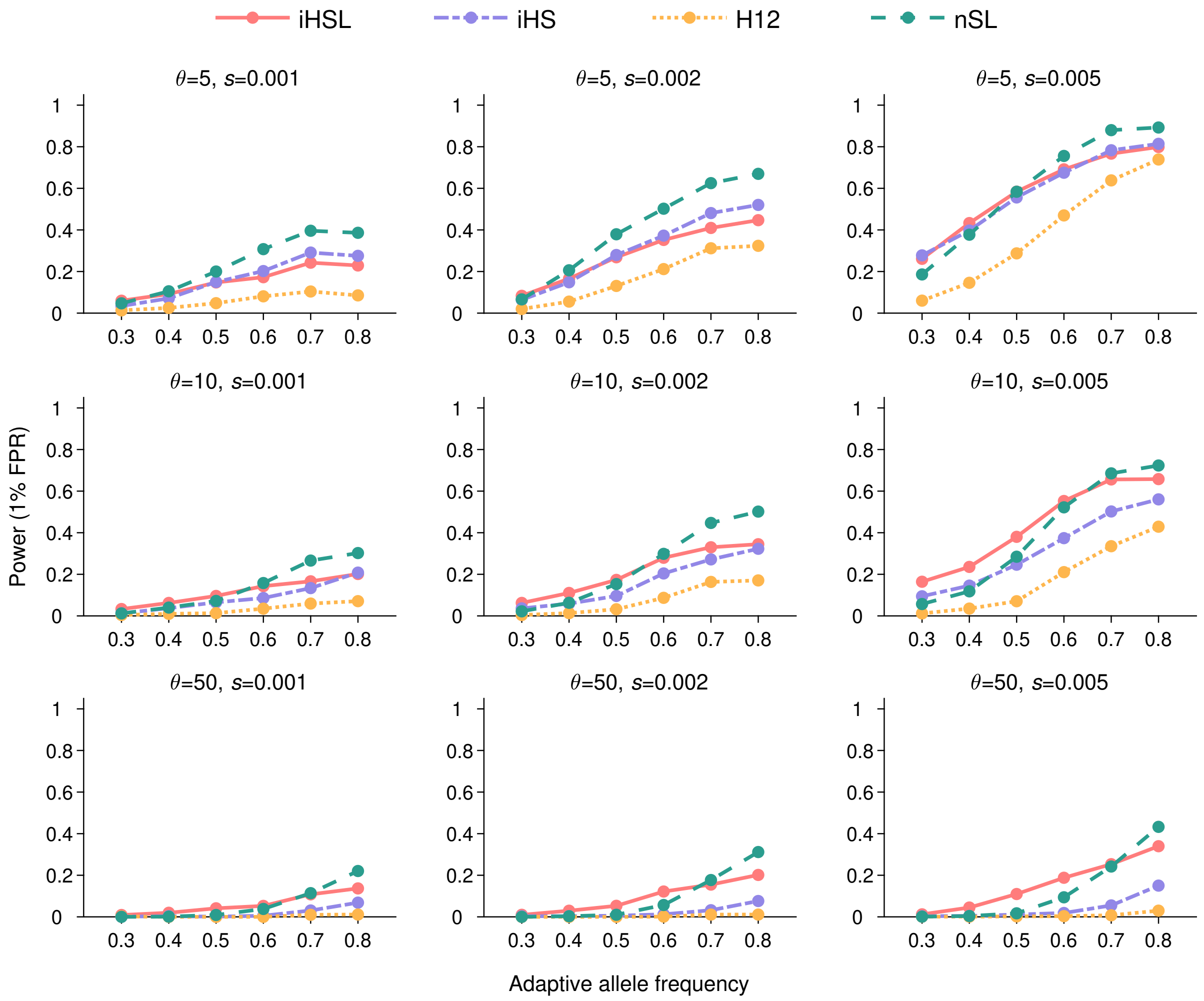

Fig. S10. Comparison of the power of four methods for detecting soft selective sweeps from recurrent mutations with a sample size of 50 haplotypes. The window size for H12 is set to 201 SNPs, which exhibited the highest mean power among seven tested window sizes ranging from 21 to 2001.

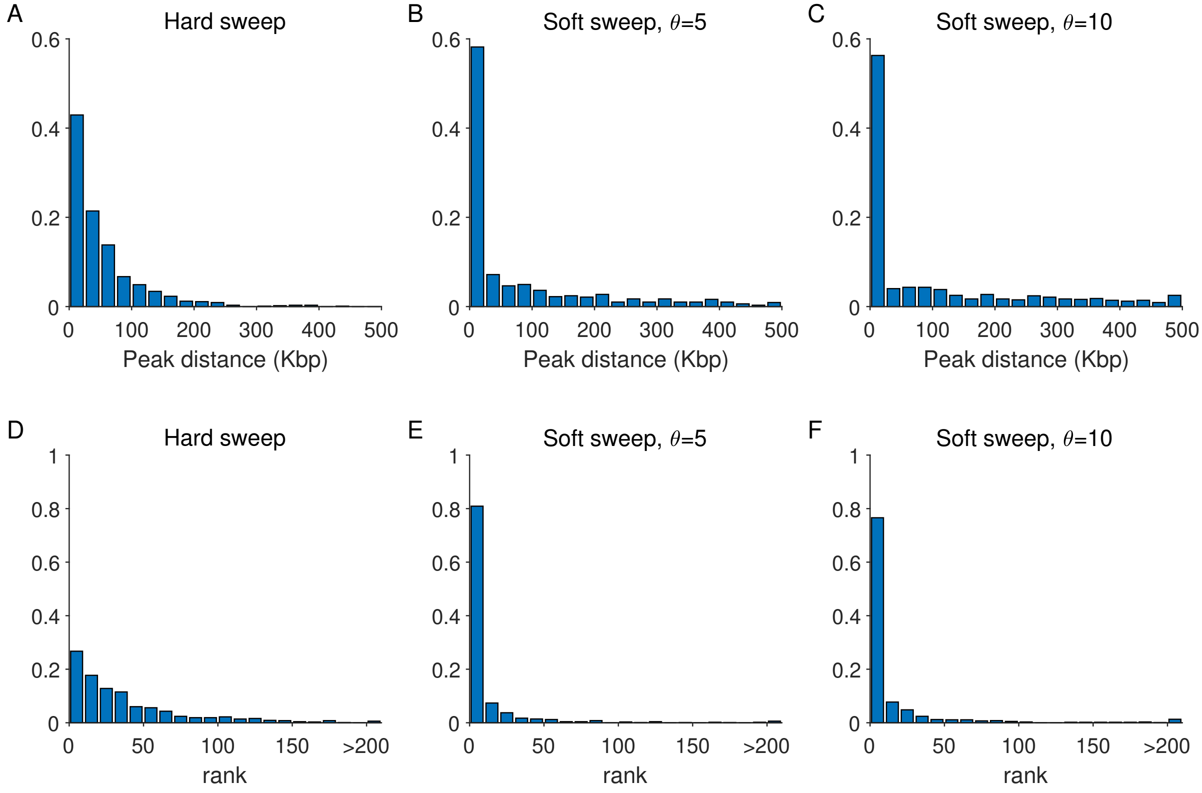

Fig. S11. HaploSweep performance in pinpointing the adaptive locus. A-C: Peak distance distributions of the adaptive locus. D-F: Rank distributions of the adaptive locus. For each panel, 1000 simulation replicates of hard or soft sweep (recurrent mutation, $\theta = 5, 10$) are generated. The iHSL values from 500 kb upstream to 500 kb downstream of the adaptive locus are calculated to obtain the peak distance and rank distributions. Simulation is carried out with the selection intensity $s = 0.02$, and the equilibrium demographic model (see *Coalescent simulation* section in the main text).

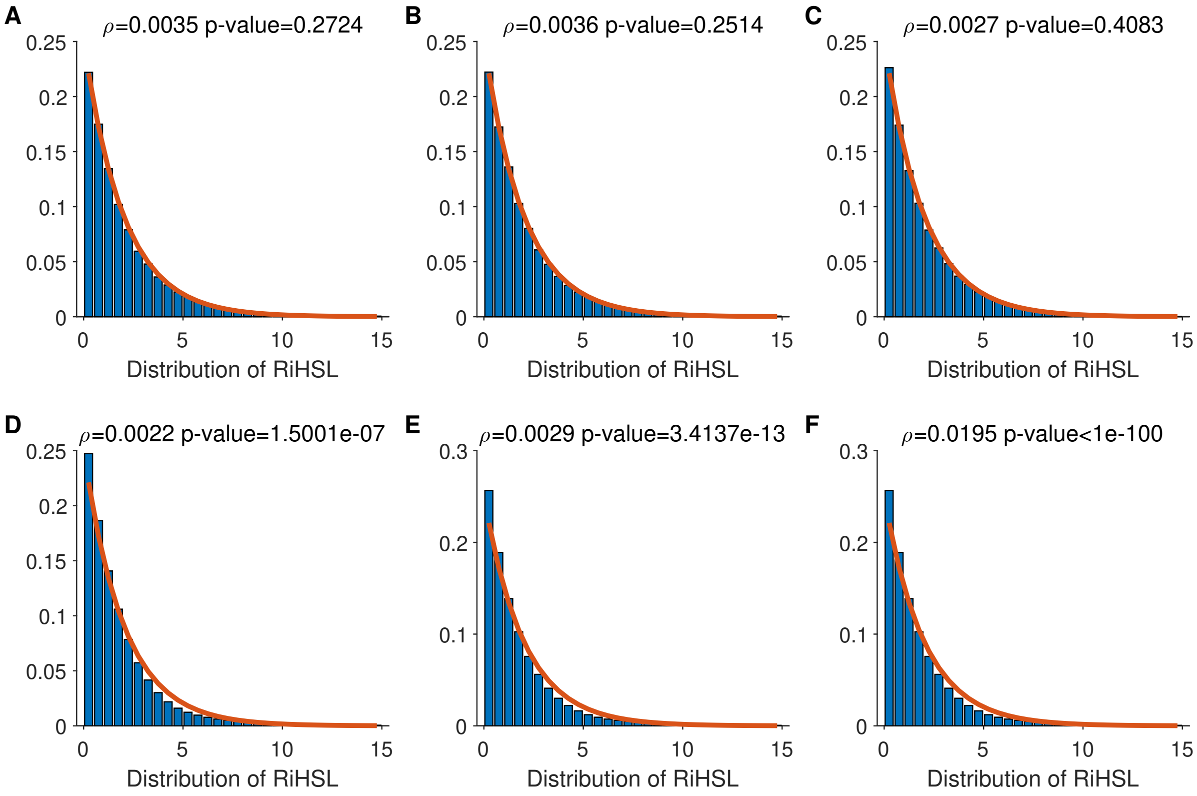

Fig. S12. Distribution of RiHSL and Pearson correlation coefficient between iHSL and RiHS. The bar charts display the distribution of RiHSL of simulated data (A, B, C) and real data (D, E, F), with the red lines representing the probability density function of the Chi-Square distribution with 2 degrees of freedom. Panels A, B, C show simulated neutral datasets for CHB, CEU, and YRI, respectively. Panels D, E, F show real data from CHB, CEU, and YRI, respectively.

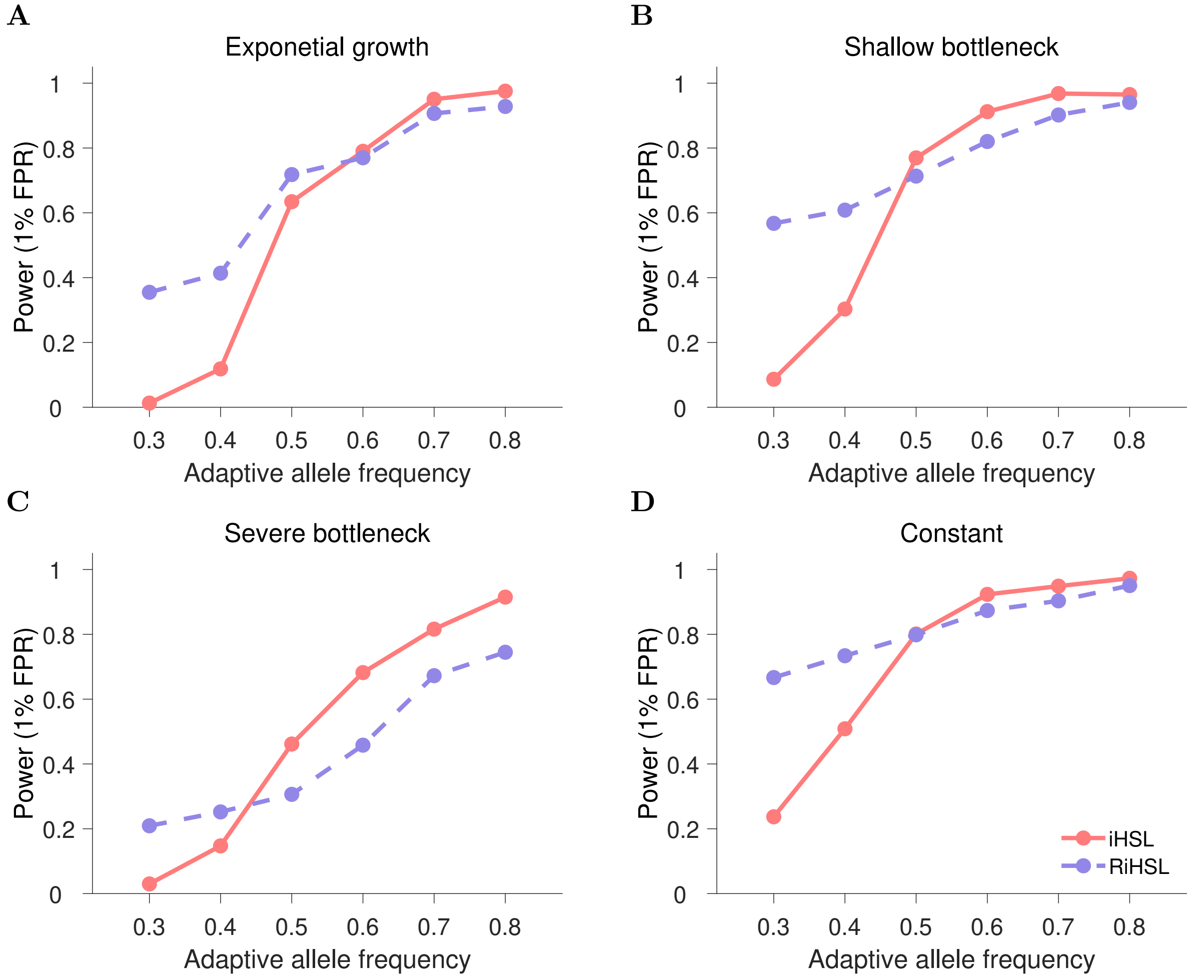

Fig. S13. Comparison of iHSL and RiHSL in detecting soft sweep signals. Selection intensity is set to $s = 0.02$, and the initial frequency is set to $f_0 = 0.1$.

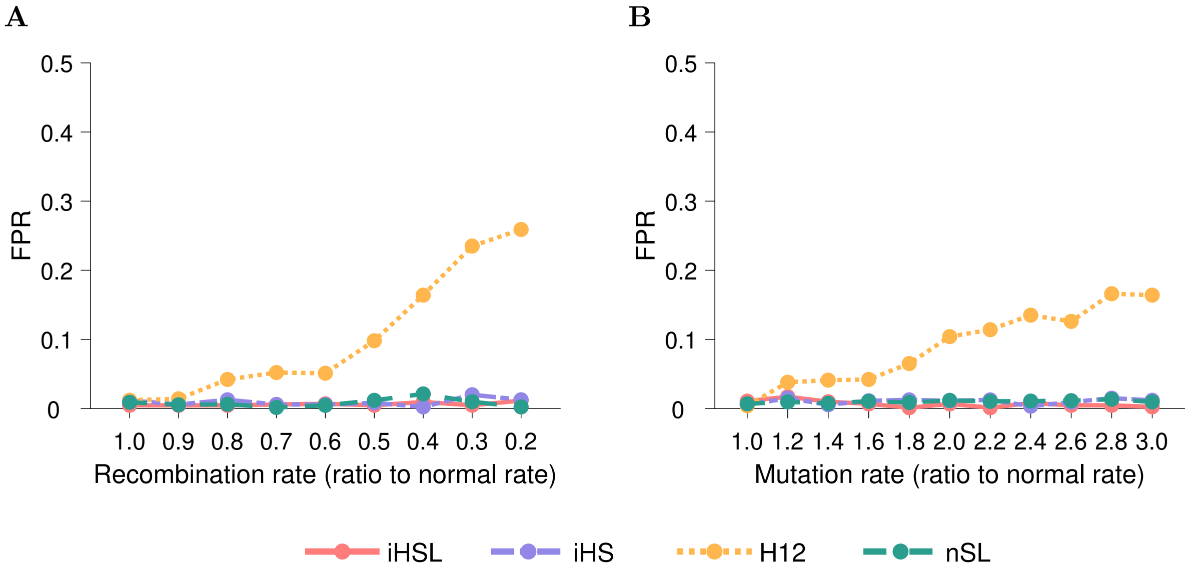

Fig. S14. False positive rates based on the heterogeneity of recombination rate and mutation rate. (A) FDR across recombination rates ranging from 0.2-1.0 times 1.25×10^{-8}; (B) FDR across mutation rates ranging from 1.0-3.0 times 2.5×10^{-8}.

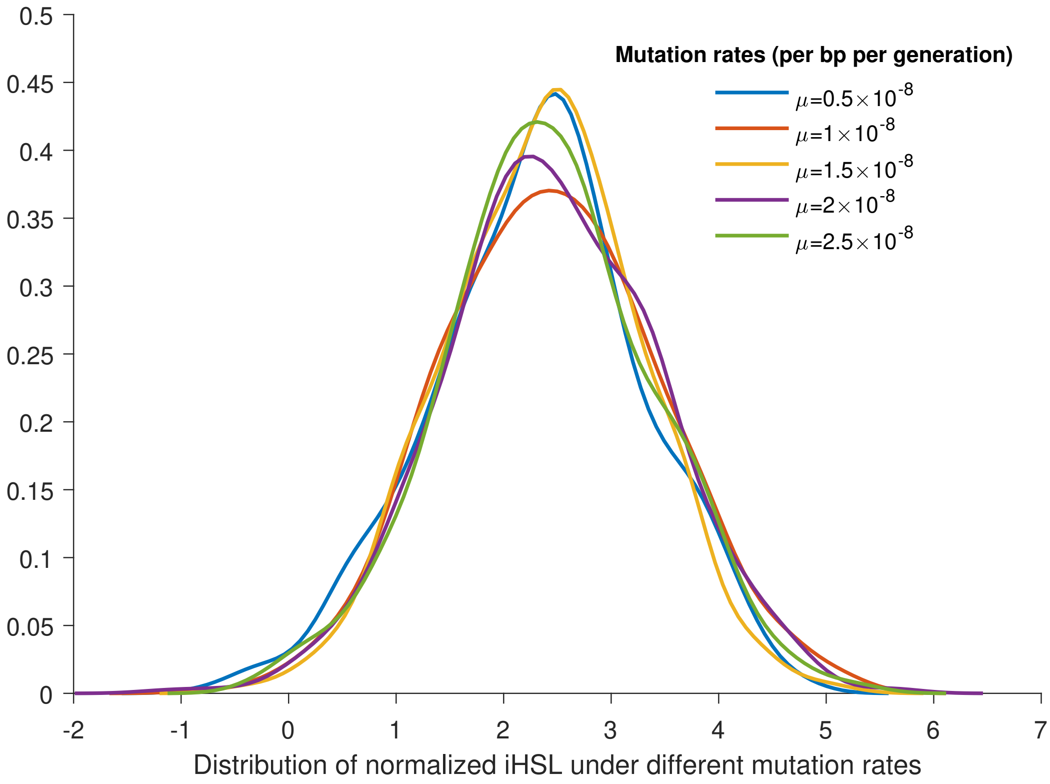

Fig. S15. Distribution of normalized iHSL values for soft selective sweeps simulated with varying mutation rates μ of 0.5×10^{-8}, 1×10^{-8}, 1.5×10^{-8}, 2×10^{-8}, and 2.5×10^{-8} per bp per generation.

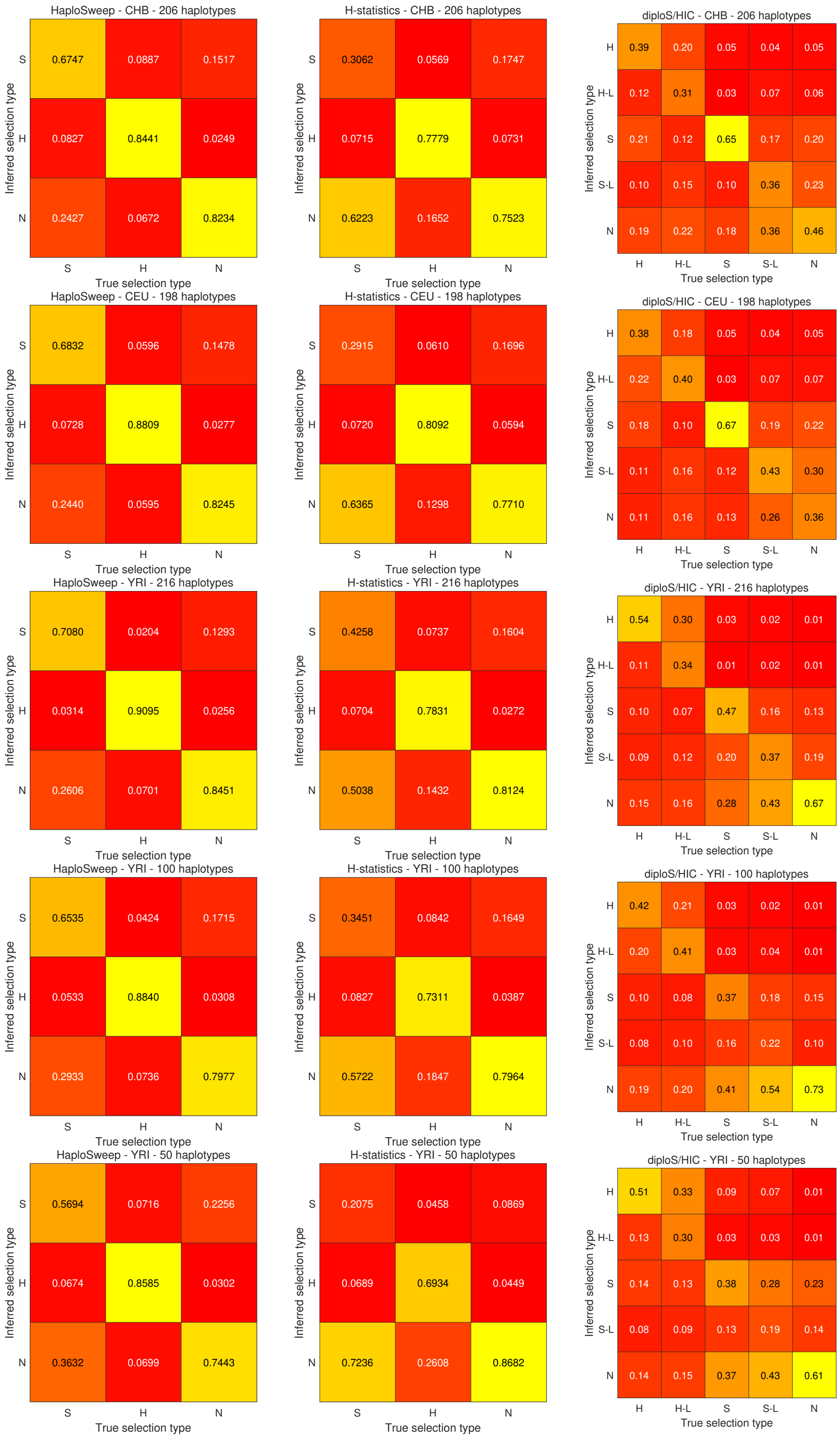

Fig. S16. Confusion matrix for HaploSweep, H-statistic, and diploS/HIC. “N” represents neutral, “H” represents hard sweep, “S” represents soft sweep, “H-L” represents hard-linked, and “S-L” represents soft-linked.

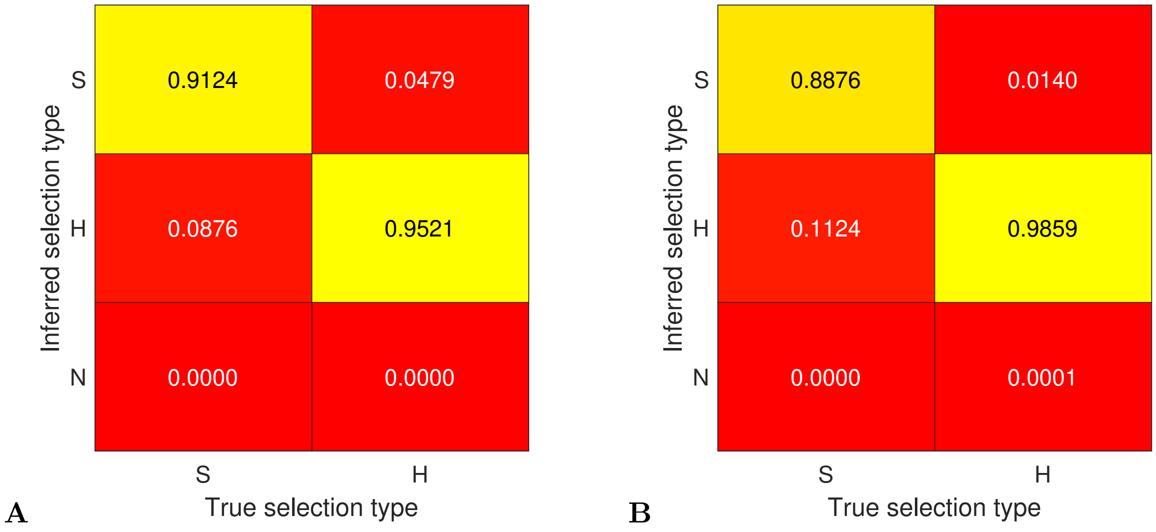

Fig. S17. The ability of classifying sweep types under mis-specified demographic history. A) classifying the sweep types of CHB assuming a demographic history of CEU. A) classifying the sweep types of CEU assuming a demographic history of CHB.

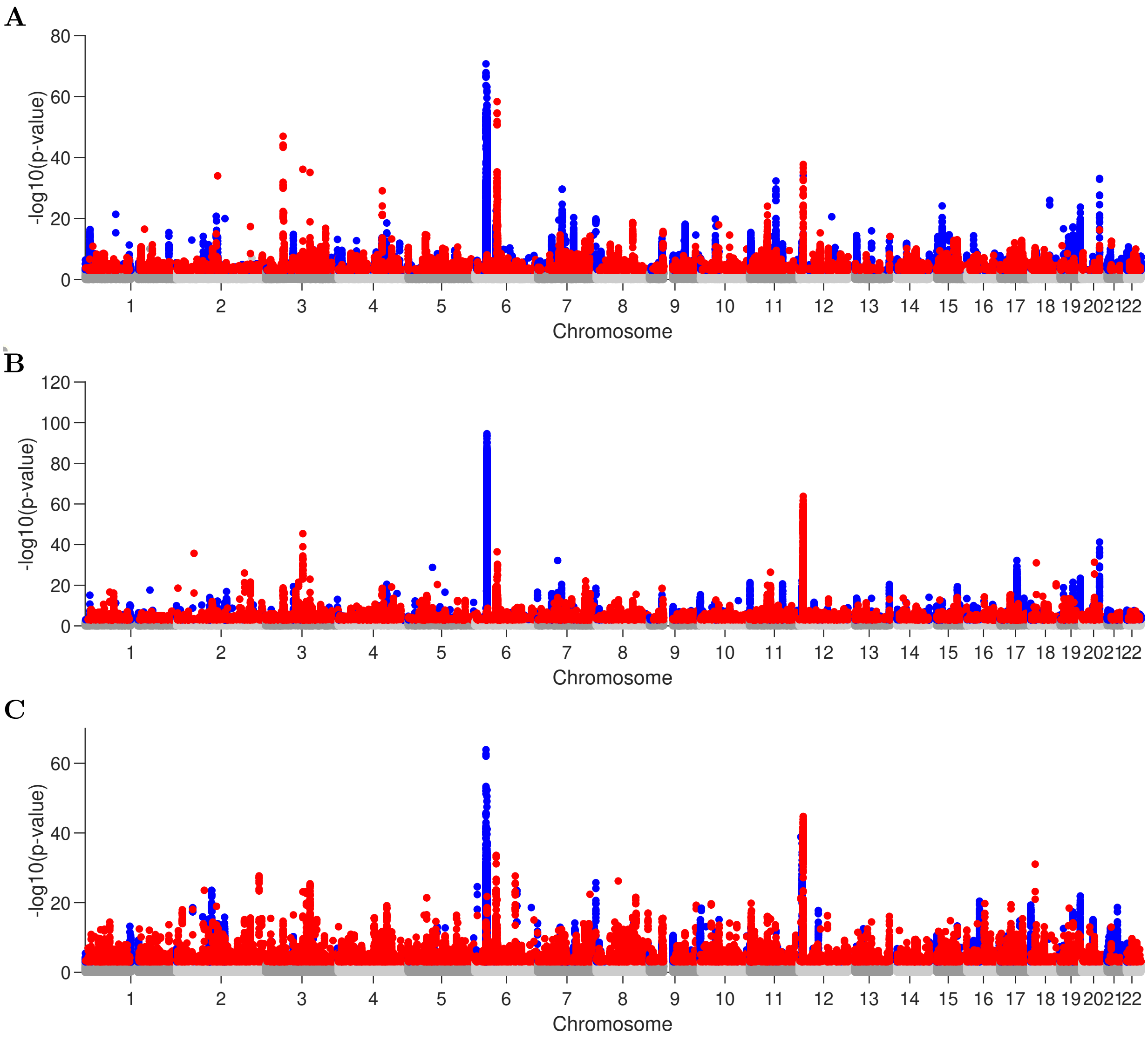

Fig. S18. Manhattan plot illustrating HaploSweep (RiHSL) P values for the three 1KGP populations: A) CHB, B) CEU, and C) YRI. Soft sweep signals are denoted with red dots, while hard sweep signals are represented by blue dots. Grey dots indicate neutral loci with p-values exceeding 0.001.

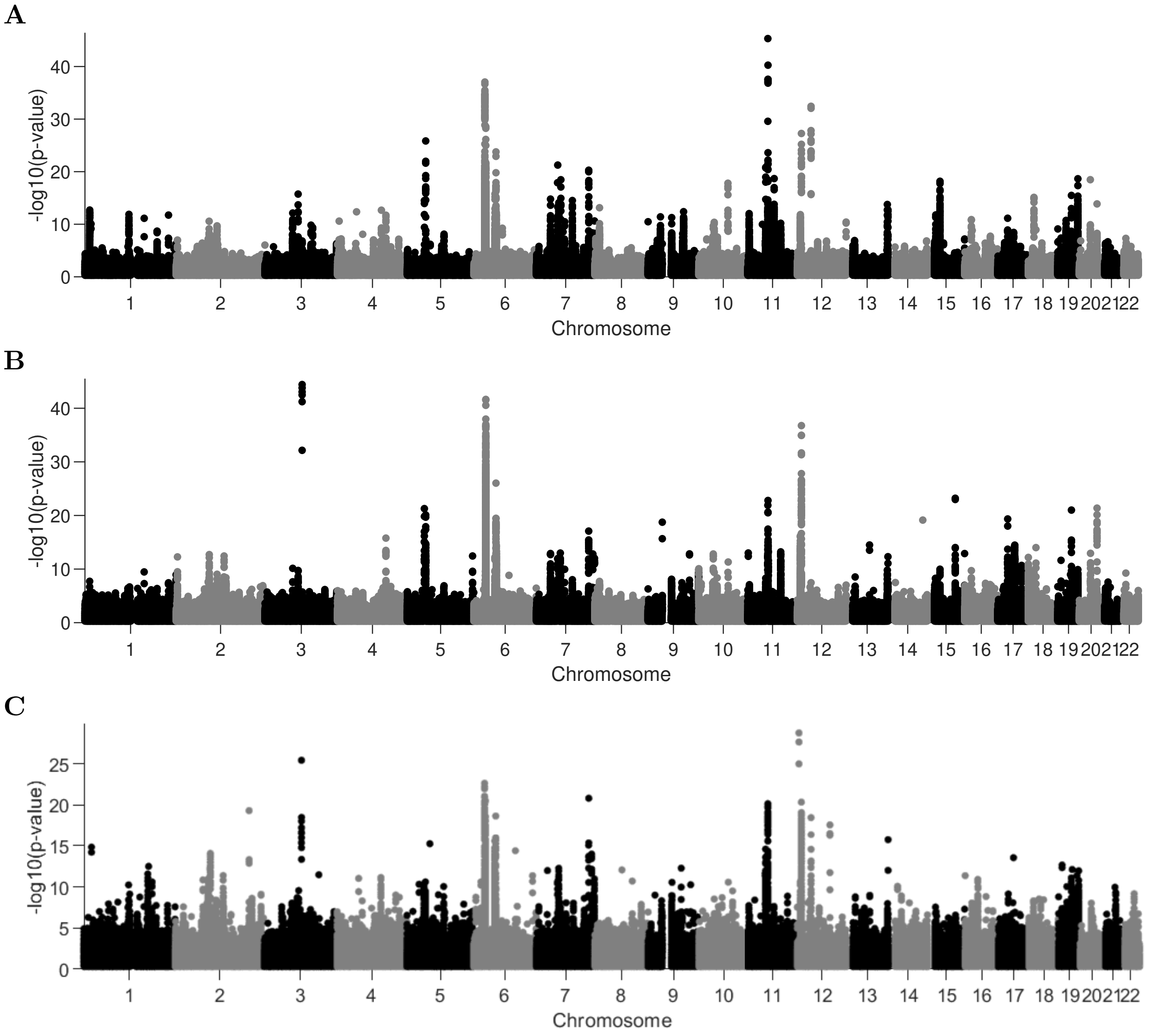

Fig. S19. Manhattan plot illustrating iHS P values for the three 1KGP populations: A) CHB, B) CEU, and C) YRI.

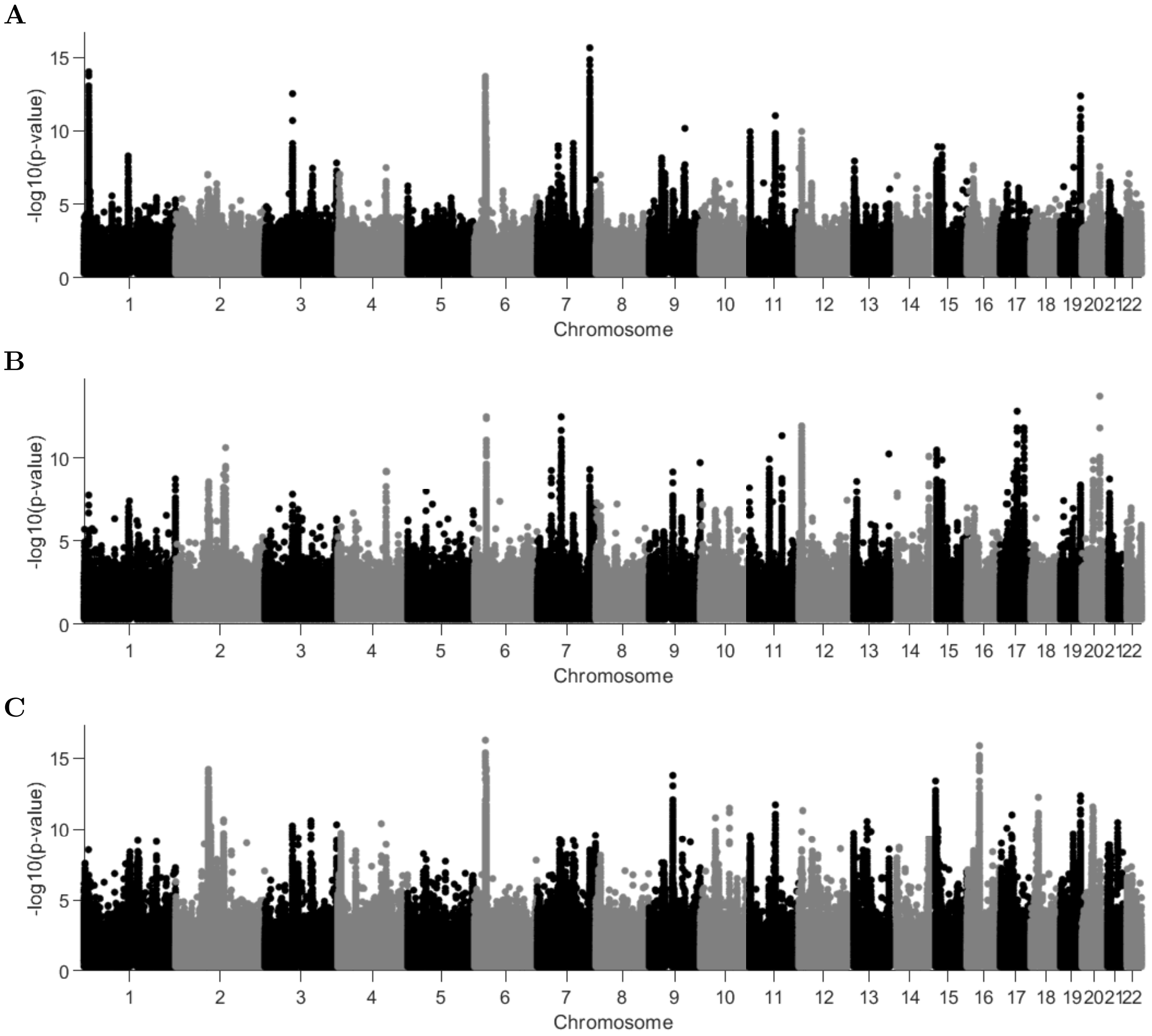

Fig. S20. Manhattan plot illustrating nSL P values for the three 1KGP populations: A) CHB, B) CEU, and C) YRI.

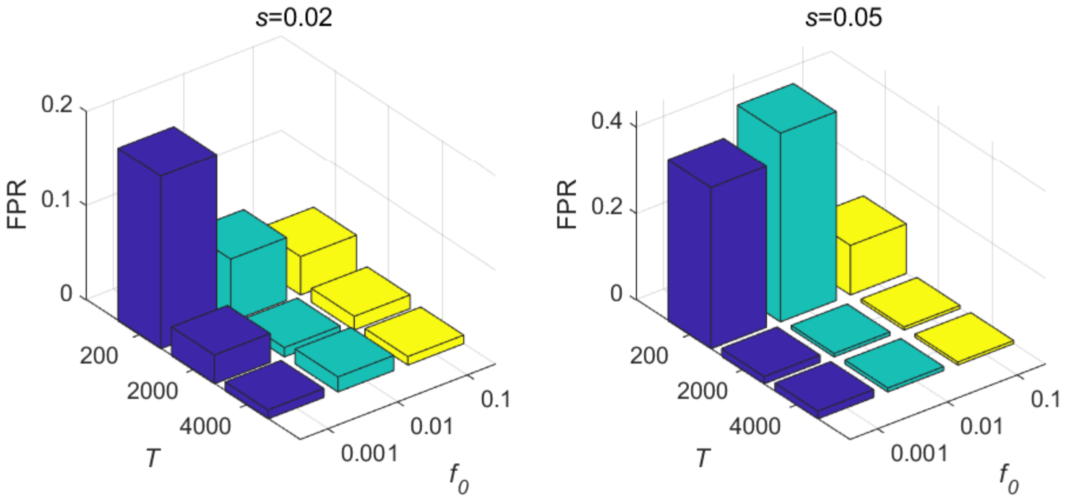

Fig. S21. Proportion of balancing selection misclassified as selective sweeps. Data is simulated under the population history of YRI (see details in the *Coalescent simulation* section of the main text). The selection intensity for heterozygotes is set to 0.02 (left panel) and 0.05 (right panel) respectively, while the selection intensities for the two types of homozygotes are 0. The initial frequencies of the adaptive allele (f_0) are set to 0.001, 0.01, and 0.1. The selection onset times (T) are set to 200, 2000, and 4000 generations.

References

Ewing, G. and Hermisson, J. 2010. Msms: a coalescent simulation program including recombination, demographic structure and selection at a single locus. *Bioinformatics*, 26(16): 2064–2065.

Gravel, S., Henn, B., Gutenkunst, R., Indap, A., Marth, G., Clark, A., Yu, F., Gibbs, R., Bustamante, C., Altshuler, D., *et al.* 2011. Demographic history and rare allele sharing among human populations. *Proc Natl AcadSci U S A.*, 108(29): 11983–11988.

Vy, H. M. T., Won, Y.-J., and Kim, Y. 2017. Multiple Modes of Positive Selection Shaping the Patterns of Incomplete Selective Sweeps over African Populations of Drosophila melanogaster. *Molecular Biology and Evolution*, 34(11): 2792–2807.
